# The upper limb Physiological Profile Assessment: Description, reliability, normative values and criterion validity

**DOI:** 10.1371/journal.pone.0218553

**Published:** 2019-06-27

**Authors:** Lewis A. Ingram, Annie A. Butler, Lee D. Walsh, Matthew A. Brodie, Stephen R. Lord, Simon C. Gandevia

**Affiliations:** 1 Neuroscience Research Australia, Sydney, New South Wales, Australia; 2 University of New South Wales, Sydney, New South Wales, Australia; 3 Platypus Technical Consultants Pty Ltd, Canberra, Australia; University of Ottawa, CANADA

## Abstract

A progressive decline in upper limb function is associated with ageing and disease. In this cross-sectional study we assessed the performance of 367 healthy individuals aged of 20 to 95 years across a battery of upper limb clinical tests, which we have termed the upper limb Physiological Profile Assessment (PPA). The upper limb PPA was designed to quantify the performance of the multiple physiological domains important for adequate function in the upper extremities. Included are tests of muscle strength, unilateral movement and dexterity, position sense, skin sensation, bimanual coordination, arm stability, along with a functional task. We report age and gender normative values for each test. Test-retest reliability ranged from good to excellent in all tests (intra-class correlation coefficients from 0.65 to 0.98) with the exception of position sense (0.31). Ten of the thirteen tests revealed differences in performance between males and females, twelve showed a decline in performance with increasing age, and eight discriminated between older people with and without upper limb functional impairment. Furthermore, most tests showed good external validity with respect to age, an upper limb functional test and self-reported function. This profiling approach provides a reference range for clinical groups with upper limb sensory and motor impairments and may assist in identifying undiagnosed deficits in the general population. Furthermore, the tests are sufficiently reliable to detect motor impairments in people with compromised upper limb function and evaluate the effectiveness of interventions.

## Introduction

The upper limbs play a critical role in everyday living. Fine motor skills are essential for self-care, including feeding, dressing and grooming. The upper limbs also contribute to gross motor skills such as crawling, walking, balance recovery, as well as physical protection when the recovery of balance is not possible [1]. Ageing is associated with a progressive decline in one or more physiological domains that are critical for adequate postural balance, including vision, muscle strength, proprioception and reaction time [2] and may be critical for upper limb function. Deficits in each of these systems are well documented in many neurological disorders such as multiple sclerosis [3], Parkinson’s disease [4,5] and stroke [6].

The sole reliance on a medical diagnosis for upper limb dysfunction may be sub-optimal due to considerable variability in the presentation and severity of symptoms of specific diseases and also because multiple comorbidities become increasing prevalent in older age [7,8]. Furthermore, sensory and motor impairments are highly prevalent in older people without documented medical conditions. A framework that quantifies an individual’s upper limb motor impairments has excellent potential to complement the ‘disease-based/medical’ model in that it could provide precise measurement of impairment levels that would be valuable for both guiding and evaluating interventions [9].

**Fig 1:**
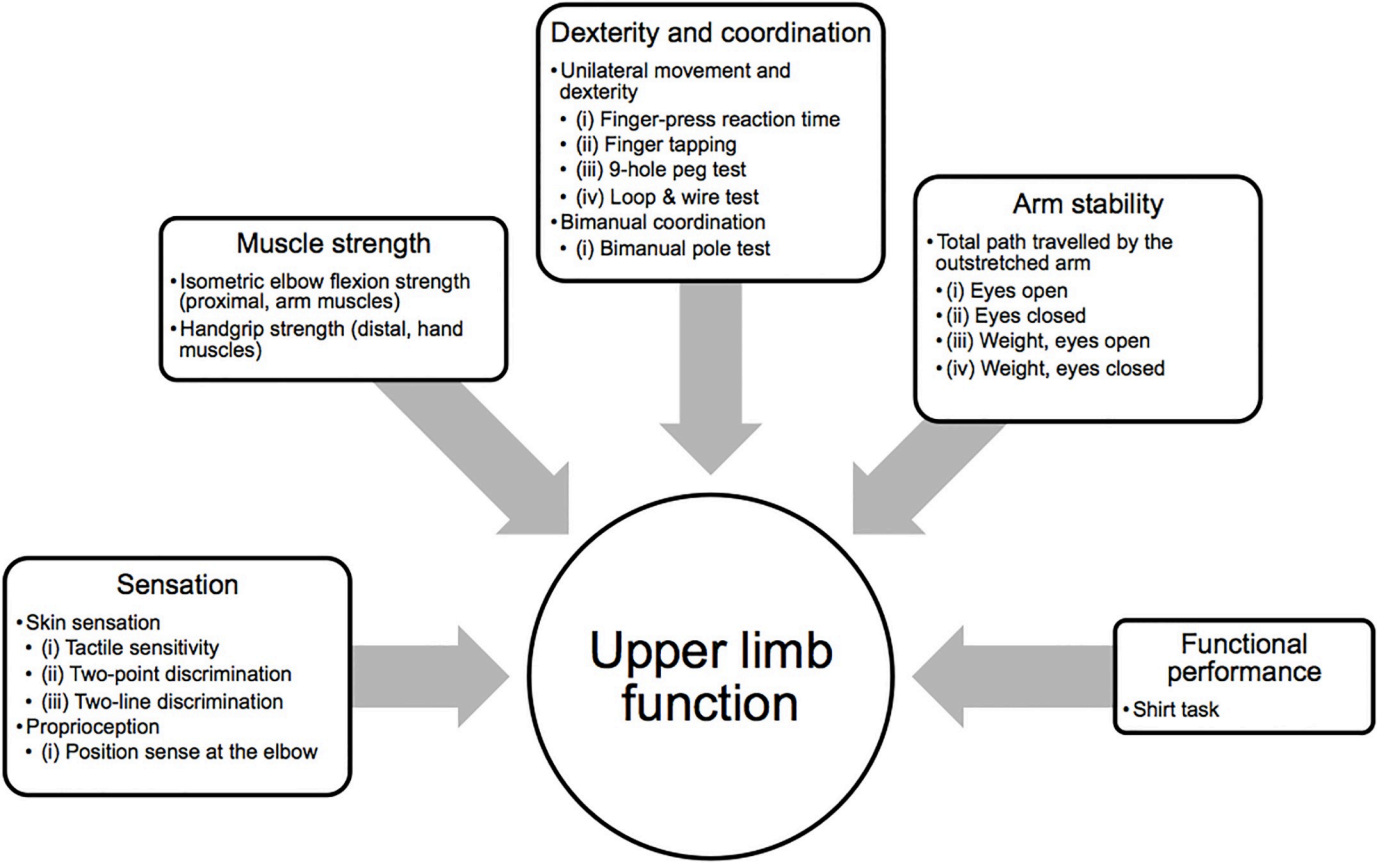
Physiological domains model. Contributory physiological domains critical for upper limb function.

Considering the hypothesis that upper limb function is derived from the complex interplay of various physiological domains, a single clinical test to quantify performance would be insufficient. Rather, a battery of tests, the upper limb physiological profile assessment (PPA), that individually measures the various contributory physiological domains is essential to assess upper limb function. This hypothesis is based on the sensitivity of the original PPA (lower limb) to identify older people at risk of falling [9,10]. The original PPA (lower limb) combined five tests of sensorimotor function into a composite fall risk score [9]. With respect to the upper limb PPA, the posited physiological domains required for upper limb function are outlined in Fig 1. Additional considerations with respect to clinical utility were that the tests needed to be: simple to administer, have short administration times, be feasible for all people to undertake, comprise low-tech, robust and portable equipment and provide quantitative, valid and reliable measurements.

The primary aim of this study was to present age and gender normative values for tests that measure muscle strength, unilateral movement and dexterity, position sense, skin sensation, bimanual coordination and stability in the upper limbs in healthy individuals across the adult lifespan. Secondary aims were a) to determine the test-retest reliability for each of the tests, b) to explore gender differences and associations with ageing and test performance, c) to determine the criterion validity of each test by assessing whether they could discriminate between people with and without self-perceived upper limb functional impairment and d) to determine how well the tests, alone and in combination, could explain the variance of a composite measure of upper limb function and e) identify potential latent factors for the test measures with a principal component analysis.

## Methods

### Participants

Three hundred and sixty seven neurologically healthy individuals over seven decades from the 20s to 80+ (20 to 95 years, 172 males and 195 females) were recruited to participate in the study, with a minimum of 20 males and 20 females from each decade. Participants were recruited from the NeuRA Research Volunteer database, staff of a large insurance and consulting company, and the local community in response to flyers placed at the University of New South Wales, the local hospital and on community noticeboards. For inclusion, prospective participants had to be aged 20 years or older, able to sit unassisted for the duration of testing, and not have any major neurological disease such as stroke, spinal cord injury or multiple sclerosis. All participants were screened to exclude participants with clinical signs of upper limb musculoskeletal or neurological deficits. Handedness was self-reported. 32 participants nominated their left hand as their dominant hand, with all remaining participants identifying as right-hand dominant. Testing took place between February 2016 and October 2017, and was conducted either at Neuroscience Research Australia, or at the participant’s home or workplace. Each participant provided written, informed consent. Ethical approval was granted by the Human Research Ethics Committee, University of New South Wales (HC 15607). All assessments were conducted in accordance with the Declaration of Helsinki (2008).

### Procedure

At the beginning of the assessment, participants completed the Disabilities of the Arm, Shoulder and Hand (DASH) questionnaire [11]. The DASH provides a valid measure of self-perceived upper-extremity function [12]. It is scored on a 100-point scale, with higher values indicative of greater levels of impairment in the upper limb. A score of >15/100 has been suggested as being discriminative between those with and without upper limb impairment [13]. DASH scores were not calculated until after the completion of testing. Visual acuity and contrast sensitivity were then screened using a Logarithmic Visual Acuity Chart (SLOAN Two Sided ETDRS Format Near Point Test) calibrated for testing at 40 cm (Precision Vision, USA) and the Melbourne Edge Test, respectively; ensuring that each participant had satisfactory vision to complete the tests [14,15]. Participants were permitted to wear their visual aids. They then completed each of the upper limb tests (see below). Test administration took approximately 60 minutes over a single visit. The initial 30 participants (21 to 81 years, 15 males and 15 females) completed the tests on a second visit, approximately one week later. Data from these participants was used to determine test-retest reliability for each test. For the reliability component of the study, n = 29 participants were assessed by the same examiner. Another examiner assessed the remaining participant, with that same examiner testing the participant at both test and re-test.

The test battery, termed the upper limb Physiological Profile Assessment (PPA), consisted of 13 individual tests (with a total of 17 outcome measures) classified into the following domains: muscle strength, unilateral movement and dexterity, position sense, skin sensation, bimanual coordination, arm stability and functional tasks (see S1 Table for rationale behind inclusion of selected tests). Each test is outlined in detail below. For clarification, we have collectively referred to each of the initial 12 tests as ‘sensorimotor tests,’ as they are purported to exclusively or mostly measure the function of a single physiological domain. The final test is referred to as a ‘composite measure,’ as it was selected to assess the function of numerous physiological domains within a single test. All tests were performed with the participant seated unless stated. Participants performed each test with their dominant hand when applicable. Five experienced examiners conducted the assessments in the main study.

### Measurements

#### Muscle strength

**Fig 2:**
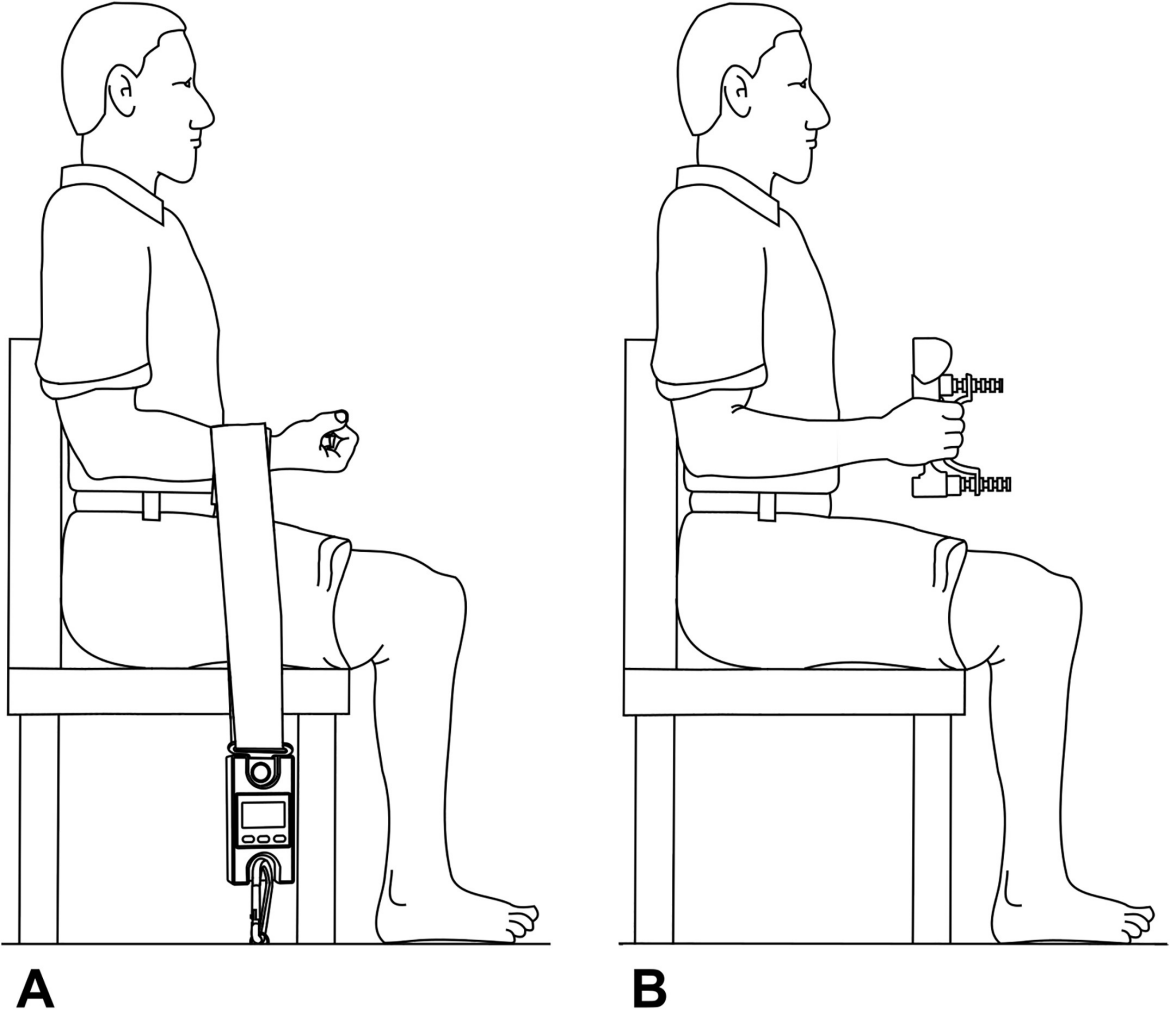
Tests of muscle strength. **(A)** Isometric elbow flexion strength. The participant pulled their arm against the Velcro strap by attempting to move their hand towards their shoulder as forcefully as they could for 2–3 seconds. **(B)** Handgrip strength. The participant squeezed the handheld dynamometer as forcefully as they could for 2–3 seconds.

Isometric elbow flexion strength. The participant sat with their upper arm by their side, elbow bent to 90 degrees and forearm supinated (Fig 2A). The custom made set-up consisted of a digital hanging scale (Scales Plus, Australia) that was fixed to a portable wooden platform that was situated underneath the chair. The Velcro strap attached to the hanging scale was firmly secured around the participant’s wrist, immediately proximal to the distal wrist crease. The examiner adjusted the tension of the strap to ensure there was no slack. When instructed, the participant pulled up against the strap by attempting to move their hand towards their shoulder as forcefully as they could for 2–3 seconds while the examiner provided verbal encouragement throughout. The examiner ensured that there were no compensatory movements in the form of trunk extension or lateral flexion away from the tested arm. The best of three trials (measured in kilograms) was recorded as the participant’s test score. Thirty seconds rest between successive trials controlled for fatigue.

Handgrip strength. Handgrip strength was assessed using a Jamar+ Digital Dynamometer (Lafayette Instrument Company, USA) [16]. The participant sat holding the dynamometer with their upper arm by their side, elbow bent to 90 degrees and forearm midway between pronation and supination (Fig 2B). When instructed, the participant squeezed the dynamometer as forcefully as they could for 2–3 seconds while the examiner provided verbal encouragement throughout. The best of three trials (measured in kilograms) was recorded as the participant’s test score. Thirty seconds rest between successive trials controlled for fatigue.

#### Unilateral movement & dexterity

**Fig 3:**
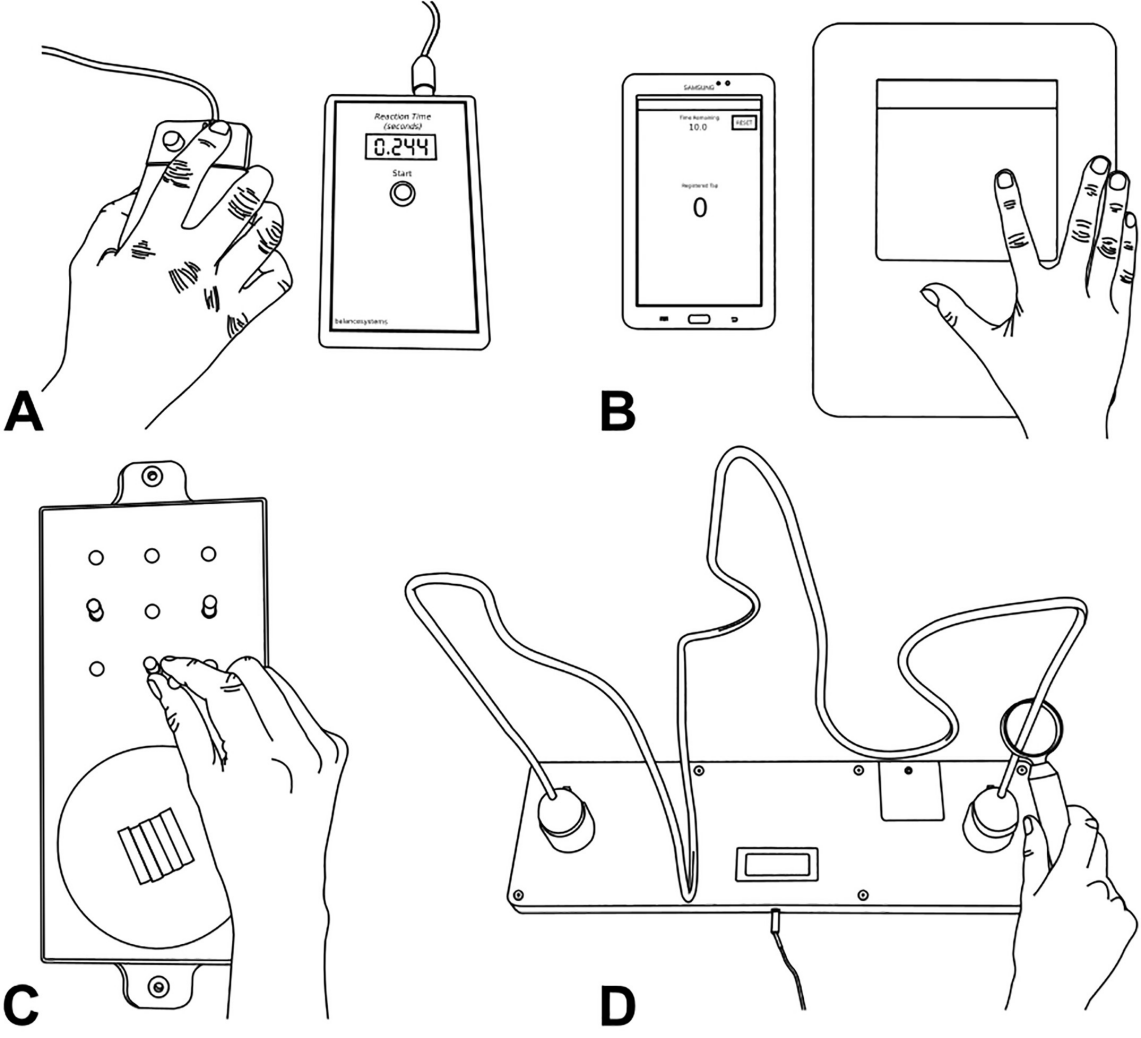
Tests of unilateral movement and dexterity. **(A)** Finger-press reaction time. The participant pressed the right button as soon as the emitting red light stimulus (embedded in the left button of the mouse) was illuminated. **(B)** Finger tapping. The participant tapped their dominant index finger up and down onto the tapping sensor as many times as they could over a 10s period. **(C)** 9-hole peg test. The participant picked up one peg at a time from the moulded dish and placed them into any of the nine holes behind the dish, before individually returning each peg back to the moulded dish as quickly as they could. **(D)** Loop and wire test. The participant held the handle attached to the ring and attempted to move the ring through the copper wire maze as fast and as accurately as possible. Two trials were completed, one in each direction.

Finger-press reaction time. Reaction time was measured using the protocol originally described by Lord et al. [17] The participant rested their dominant index finger over the right button of a modified computer mouse, which was connected to an electronic timer (Fig 3A). The participant focused their attention on the red light emitting diode (LED) embedded in the left button of the mouse, pressing the right button as soon as the LED was illuminated. The electronic timer recorded the duration between the light stimulus and participant’s response in milliseconds. The examiner pressed the ‘start’ button on the electronic timer to commence the next trial. A built-in variable delay of 1–5 seconds eliminated potential cues that may assist the participant each time the examiner pressed the ‘start’ button. Five practice trials, followed by 10 experimental trials were performed, with the average of the latter calculated as the test score (measured in milliseconds).

Finger tapping. The finger tapping test was modelled on the widely used and reported test of motor function (for review, see ref [18]). The test measured the number of times the participant could tap their dominant index finger up and down over a 10-second period. Each tap was recorded by a tapping sensor (Magic Trackpad, Apple Inc., USA), which was synced to a Samsung Galaxy Tab 3 (using a simple custom made Finger Tap Counter application). The participant placed the tip of their index finger lightly on top of the tapping sensor, with the thumb and remaining fingers resting either side of the sensor (Fig 3B). Ensuring that each tap was isolated to the metacarpophalangeal joint (i.e. knuckle), the participant tapped their index finger as many times as possible for a trial time of 10-seconds. The 10-second countdown period commenced with the first tap of the sensor. The participant’s test score was the number of taps completed in the 10-second trial, recorded and displayed on the Samsung Galaxy Tab 3 via the Finger Tap Counter application.

9-hole peg test. The 9-hole peg test (9-HPT) is used extensively in research and the clinical setting as a measure of finger dexterity [19,20]. The Roylan 9-HPT board was placed on top of a non-stick mat on the table in front of the participant with the long-axis of the board perpendicular to the participant’s midline. The participant rested their dominant hand on the table in front of the moulded dish on the board containing the nine plastic pegs (Fig 3C). Following a partial demonstration by the examiner, the participant commenced the test by picking up one peg at a time and placing it into any of the nine holes behind the dish (order of placement was not prescribed). Each peg was then individually returned to the dish before the test was completed. Participants were asked to perform the test as quickly as they could in a single trial. Time to complete the test (contact with the first peg to return of the last peg to the dish and measured in seconds) was recorded.

Loop and wire test. The custom made loop and wire test was designed to measure dexterity of the upper limb as the participant navigates a hand-held ring through a three-dimensional maze (Fig 3D). The loop and wire apparatus was positioned approximately 25 cm from the edge of the table in front of the participant. Following an initial half-length practice trial, the participant held the handle attached to the ring and attempted to move the ring through the copper wire maze as fast *and* as accurately as possible, i.e. without touching the ring on the copper wire. An electronic timer was initiated once the participant commenced the test, stopping when the ring was placed in the holder at the opposite end of the maze. Two trials were performed, one in each direction. Right-handed participants moved right-to-left, then left-to-right. The order was reversed for left-handed participants. The total number of touches was recorded and displayed on an LCD screen at the completion of each trial. The total number of touches was averaged across both trials to give the participant’s test score.

#### Position sense

**Fig 4:**
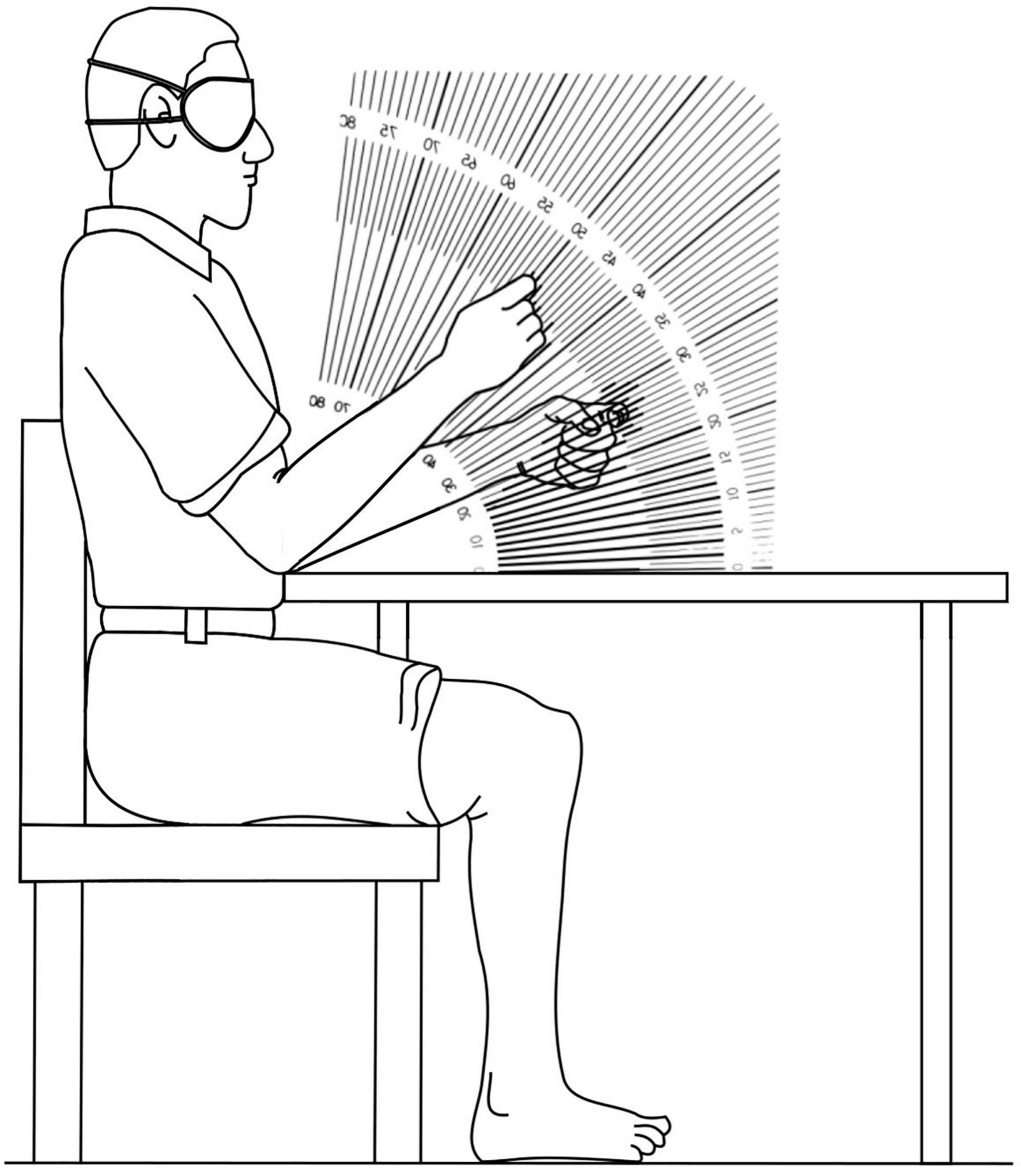
Position sense. The blindfolded participant attempted to match the position of their non-dominant index finger (passively positioned by the examiner) with their dominant index finger by bending their elbow. For each trial, the difference in degrees between both index fingers was recorded. The average error in absolute terms of the five trials was then recorded as the participant’s score.

Position sense. Position sense was measured using a modified protocol of that originally described by De Domenico and McCloskey [21]. A protractor marked on a clear acrylic sheet was positioned on the table perpendicular to the participant’s midline (Fig 4). With both forearms resting on the table either side of the protractor, the blindfolded participant held a ‘trigger’ posture by pointing both index fingers inwards as the examiner passively moved the non-dominant hand to place the index finger at five predetermined angles (50°, 70°, 30°, 40°, 60°, presented in the same set order for every participant) on the protractor. The participant attempted to match the position with their dominant index finger by bending their elbow. The examiner recorded the difference between the tips of both index fingers to the nearest degree. The participant was then instructed to relax by returning both forearms back to the table before the next trial commenced. Two practice trials were performed in the range of 30° to 70° to familiarise the participant prior to 5 experimental trials. The average error of the 5 trials was recorded as the participant’s test score (measured in °).

#### Skin sensation

**Fig 5:**
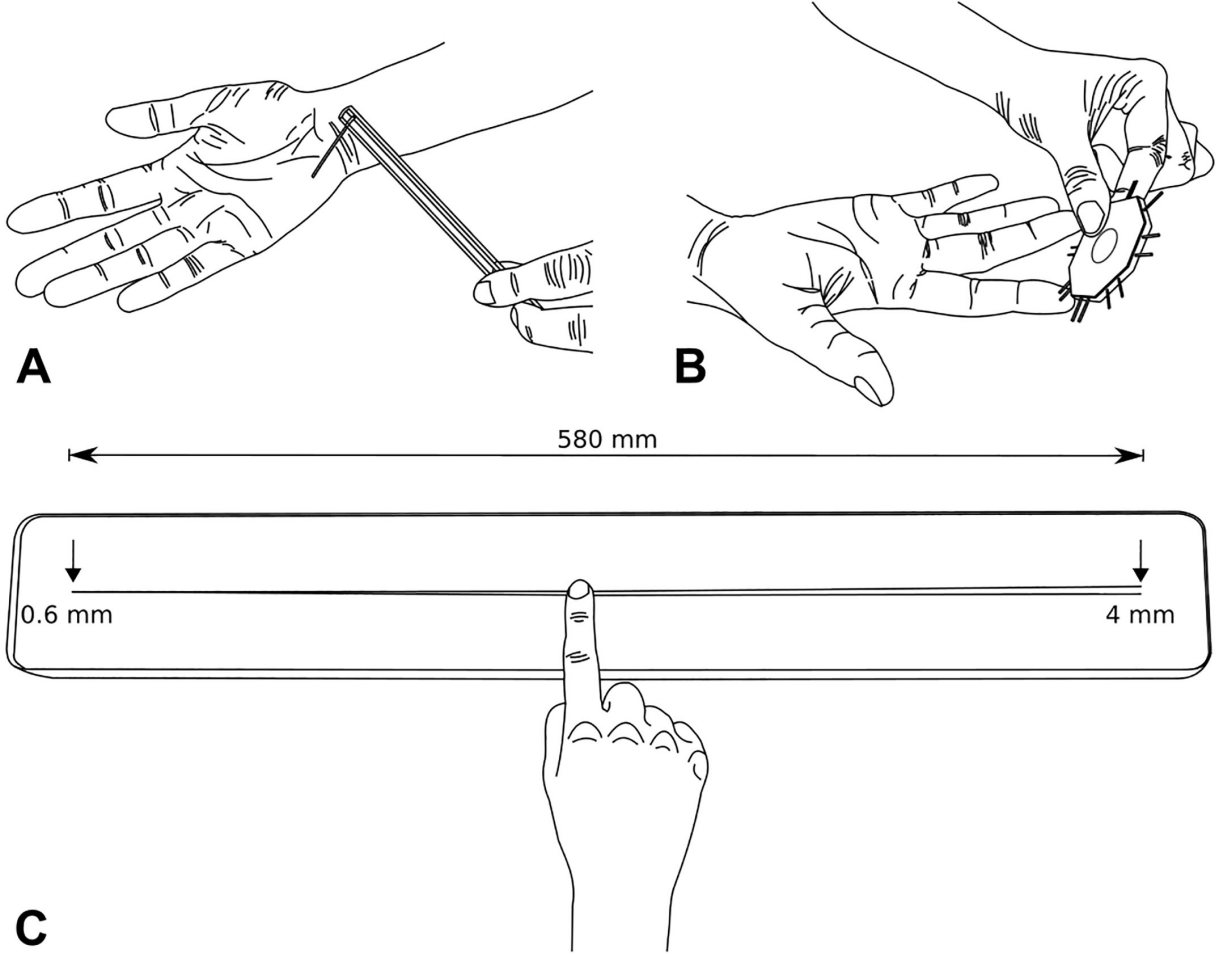
Tests of skin sensation. **(A)** Tactile sensitivity. The examiner pressed the von-Frey filament stimulus onto the blindfolded participant’s hypothenar eminence. The examiner progressively reduced the diameter of the filaments until the participant could no longer detect the stimulus. **(B)** Two-point discrimination. The blindfolded participant nominated whether they perceived one or two points at the distal tip of their index finger as the examiner randomly alternated between both options. The examiner progressively narrowed the stimulus to the point where the participant was unable to differentiate between one or two points. **(C)** Two-line discrimination. The blindfolded participant pushed down lightly and moved the distal tip of their index finger towards the right along the two ‘lines’ at a constant speed, stopping immediately when they perceived two ‘lines’ instead of one. Using a custom scale ruler (ranging from 0.6 to 4.0 mm over the 580 mm length of the two ‘lines’), the examiner records the exact spacing between the two ‘lines’ (mm) before repositioning the participant’s index finger at the right end of the board. The participant then repeated the test in the opposite direction, sliding from right to left until they felt one ‘line’ instead of two.

Tactile sensitivity. Calibrated von-Frey filaments (North Coast Medical, USA) were used to measure perceptual thresholds to cutaneous stimuli [22–24]. The filament set comprised 20 individual filaments of equal length but varying diameter. Each filament was calibrated to buckle at a specific force (measured in grams), ranging from 0.008 g to 300 g. The filaments were progressively applied to the blindfolded participant’s hypothenar eminence (Fig 5A). The hypothenar eminence shows greater sensitivity to age-related changes when compared to other sites on the palm of the hand [24], and is not confounded by subclinical carpal tunnel syndrome. A forced-choice paradigm was used whereby the participant must nominate whether they perceive the stimulus when the examiner says “A” or “B.” Using a staircase technique, the examiner started with a supra-threshold filament (the 1 g filament) before progressing towards the smaller filaments to the point where the participant could no longer detect the stimulus. (Only one participant was unable to detect the 1 g filament—in this case, the filaments were incrementally increased until the stimulus was detected.) The size of the filament was incrementally increased until detected correctly by the participant to confirm their threshold. The participant was required to identify correctly two out of three stimuli presented at each level to progress [17]. The test score was the calibrated force (measured in grams) of the smallest filament correctly identified.

Two-point discrimination. Static two-point discrimination was measured using a small- (2–8 mm) and large-interval (9–20 mm) Mackinnon-Dellon Disk-Criminator (US Neurologicals, USA) applied in a mediolateral orientation to the distal pad of the dominant index finger (Fig 5B). Unlike cutaneous sensitivity, two-point discrimination is less able to detect differences in sensitivity on different sites on the hand [24]. A forced-choice paradigm was used whereby the blindfolded participant nominated whether they perceived one or two points as the examiner pseudo-randomly alternated between both options. Care was taken to ensure that both tips touched the participant’s index finger at the same time, with the same force. Using a staircase technique, testing commenced at a supra-threshold distance before progressively narrowing to the point where the participant was unable to differentiate between one or two points. The interval between the two points was then incrementally increased to verify the participant’s two-point discrimination threshold. The participant was required to correctly identify two out of three stimuli presented at each level to progress. The test score was the smallest interval distance (measured in mm) that was correctly identified.

Two-line discrimination. The custom made two-line discrimination test was designed as an adjunct to the two-point test counterpart in response to concerns about the precision of the former to measure tactile spatial acuity [25]. The test measures the smallest distance that the participant can detect between two distinct ‘lines’ as they slide their index finger along two cords—each composed of a 0.6 mm diameter carbon fibre rod with a circular cross-sectional area that were fixed into a groove on the test board. The chords are initially positioned 0.6 mm apart before progressively diverging to 4 mm apart over a total length of 580 mm when moving from left to right (Fig 5C, see S1 Fig for apparatus specifications).

Following a short demonstration and a practice trial on a specifically designed practice board (consisting of 2 x a single line, 1 x two lines spaced 3 mm apart, and 1 x two lines spaced 6 mm apart), the participant was blindfolded before the practice board was substituted for the test board (note: the participant did *not* see the test board until the completion of testing on their second visit). The examiner passively positioned the tip of the participant’s index finger at the left end of the test board where the two ‘lines’ were positioned together. Pushing down *lightly* (described by the examiner as “firm enough to easily feel the line,” but “not too firm such that the nail bed of the finger changes colour”) and moving at a *constant speed* (demonstrated by the examiner as approximately 5 cm per second), the participant slid their finger towards the right along the two ‘lines’ (which progressively became further apart), stopping immediately when they perceived two ‘lines’ instead of one. A custom scale ruler (ranging from 0.6 to 4.0 mm over the 580 mm length of the two ‘lines’) was used to measure the exact spacing between the two ‘lines’ (in mm). The examiner then repositioned the finger at the two ‘lines’ on the right end of the board (which were separated by 4 mm) before the participant repeated the test in the opposite direction, sliding from right to left until they *felt* one ‘line’ instead of two. The custom scale ruler was once again used to measure the exact spacing between the two ‘lines.’ If the participant reached the end of the ‘lines’ before stopping, a maximum score of 4.0 mm was recorded. This protocol was completed three times, the examiner shifting the board approximately 20 cm to the left and 20 cm to the right for the second and third trials respectively. The first trial in each direction was excluded from analysis. The participant’s test score was calculated as the average of the average second and third trial scores in each direction (measured in mm).

#### Bimanual coordination

**Fig 6:**
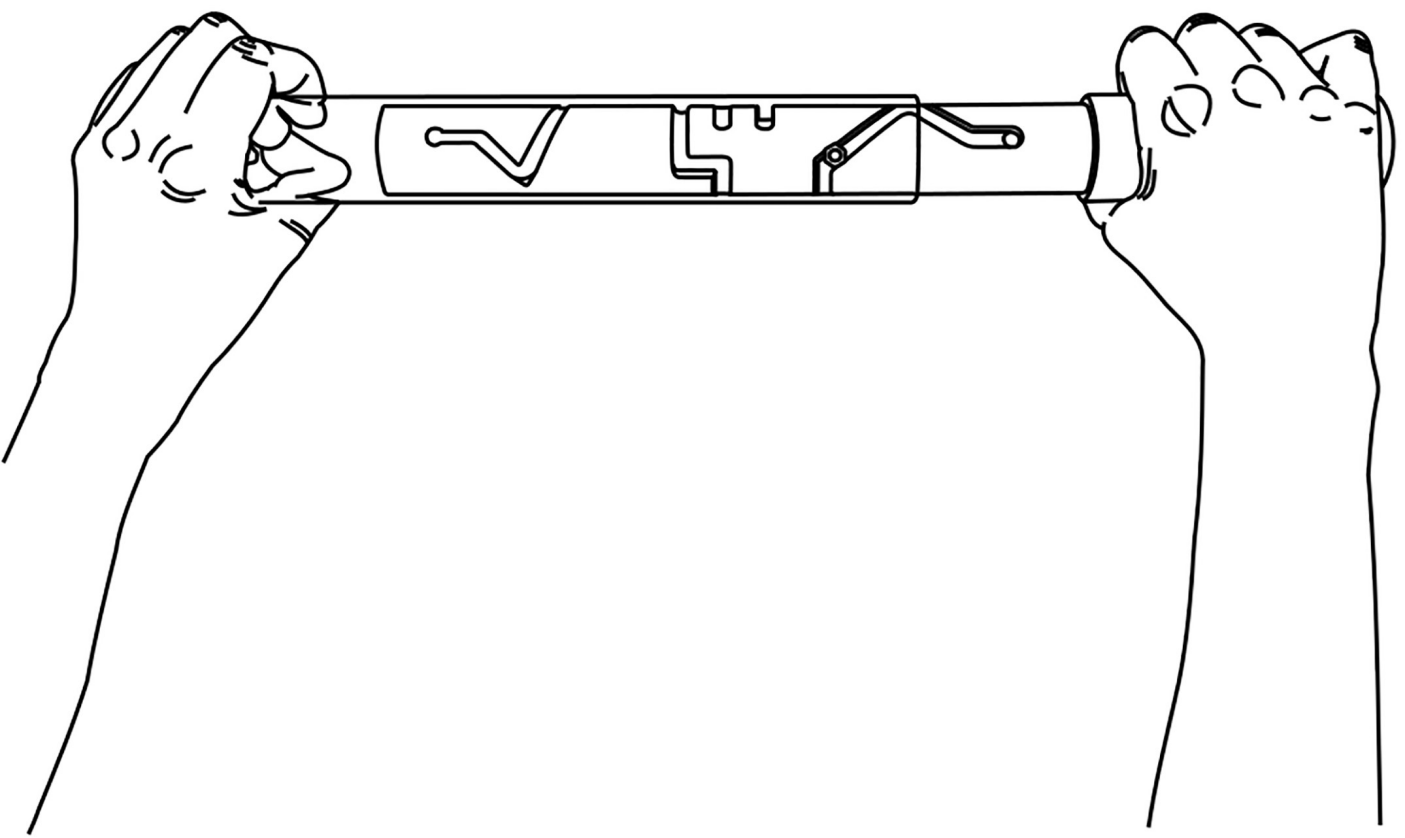
Bimanual coordination. Bimanual pole test. Holding the swivel stick with one hand at each end, the participant moved through the maze as fast as possible by flexing and extending their wrists in a coordinated manner. The time taken (in seconds) to move the screw from right-to-left and return was recorded as the test score.

Bimanual pole test. The custom made bimanual pole test was designed to measure the ability to coordinate both hands in a manipulation task. The apparatus consisted of two cylindrical-shaped pieces of Perspex—one opaque and the other clear—with the ‘former’ fitted within the inner circumference of the latter (see S2 Fig for apparatus specifications). The inner opaque cylinder contained a maze (414 mm in length) in which a screw, fixed to the surface of the outer clear cylinder, was attached. The participant held the device with one hand at each end akin to holding the handles of a rolling pin (Fig 6), the opaque end held in the right hand. To complete the test, the participant moved the screw through the maze (which contained two dead ends) as fast as possible by flexing and extending their wrists in a coordinated manner while concurrently moving the cylinders apart on the way out, then moving them together on the return. The time taken (in seconds) to move the screw from right-to-left and return was recorded as the participant’s test score.

#### Arm stability

**Fig 7:**
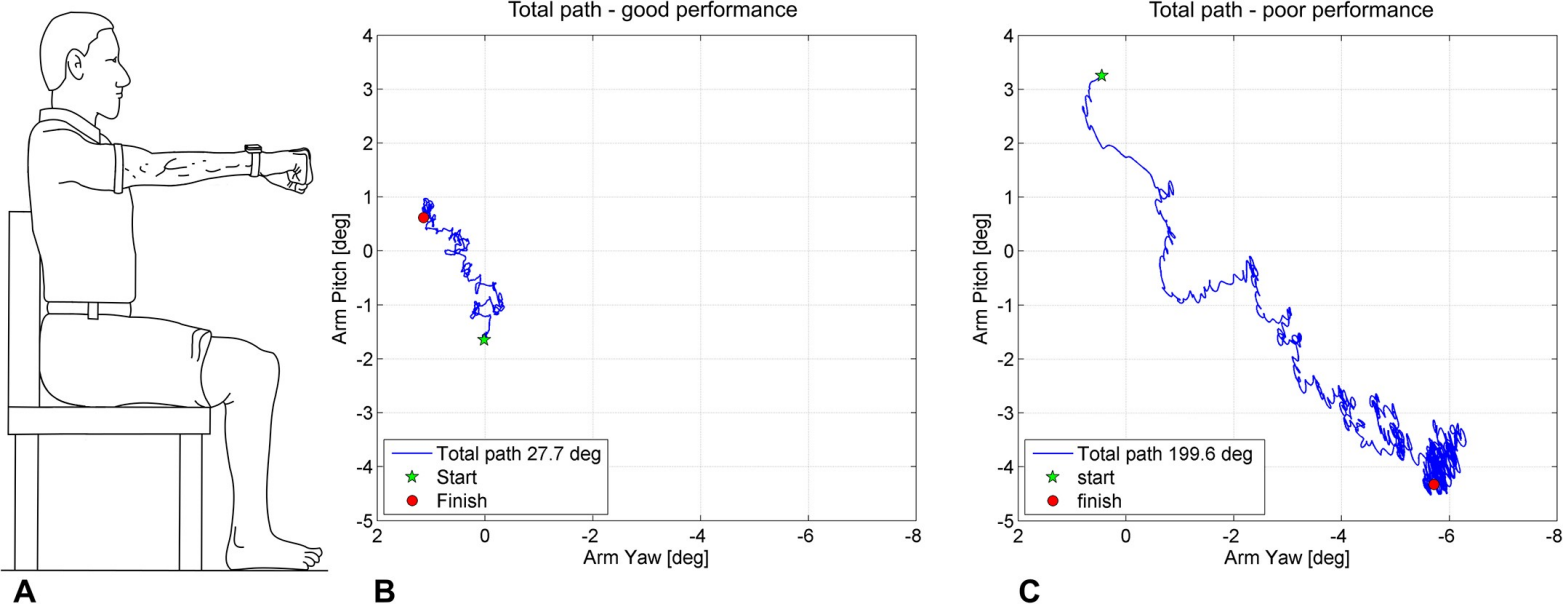
Arm stability. **(A)** The participant raised their straight arm until it was parallel to the floor while holding a small 250 g weight in their hand. The participant was instructed to hold the arm as steady and still as possible for 30 seconds. Total path (°) was recorded by an inertial measurement unit, which was attached to the wrist. **(B)** Total path (27.7°) from a 30-year-old female study participant in the weight eyes open (WEO) test condition, projected onto a yaw/pitch axis for visualisation. **(C)** Total path (199.6°) from a 95-year-old female study participant in the WEO test condition.

Arm stability. The novel arm stability test was designed to capture the ability to hold the outstretched arm still and steady for a 30s period (Fig 7A). An inertial motion unit (IMU) containing a triaxial accelerometer, gyroscope and magnetometer (OPAL by ADPM, USA, sampling frequency 128Hz) was fixed to the participant’s wrist with a Velcro strap immediately proximal to the distal radioulnar joint. Data were acquired in Motion Studio and arm movements calculated using a customised MATLAB script. The participant sat in a chair directly facing a blank wall with both feet relaxed on the ground and back firmly up against the backrest of the chair. They then raised their straight dominant arm until it was parallel to the floor. The participant was instructed to hold their arm as still and steady as possible for 30 seconds. A short rest of approximately 30 seconds followed the completion of the initial trial before the procedure was repeated another three times under the following conditions; eyes closed (blindfolded), eyes open while holding a 250 g weight in their hand, and eyes closed (blindfolded) while holding a 250 g weight in their hand. 250 g was selected as an appropriate weight as it represents a weight that would be frequently lifted when performing basic daily activities (i.e. soap, a bottle of shampoo), but not too heavy to preclude weaker participants from completing the tests. The total path (measured in degrees) was calculated from the IMU data (described in the following paragraph) and recorded as the participant’s test score for each of the four conditions.

Total path (in degrees) was calculated as the changing three-dimensional orientation of the arm about the anteroposterior (roll—pronation/supination), mediolateral (pitch—flexion/extension) and vertical axes (yaw—horizontal adduction/abduction). With respect to visualisation, these arm movements were projected onto the yaw/pitch axes (Fig 7B and 7C). Changes in arm orientation were primarily calculated from the device’s gyroscope data, which were low-pass filtered at 25Hz (with a bidirectional 4th order Butterworth filter) prior to integrating with respect to time. The accelerometer and magnetometer data were used to correct for accumulated orientation errors using a previous method specifically adapted for this study to measure arm stability [26] (see S1 File for MATLAB code used to calculate total path and example sensor data).

#### Functional performance

**Fig 8:**
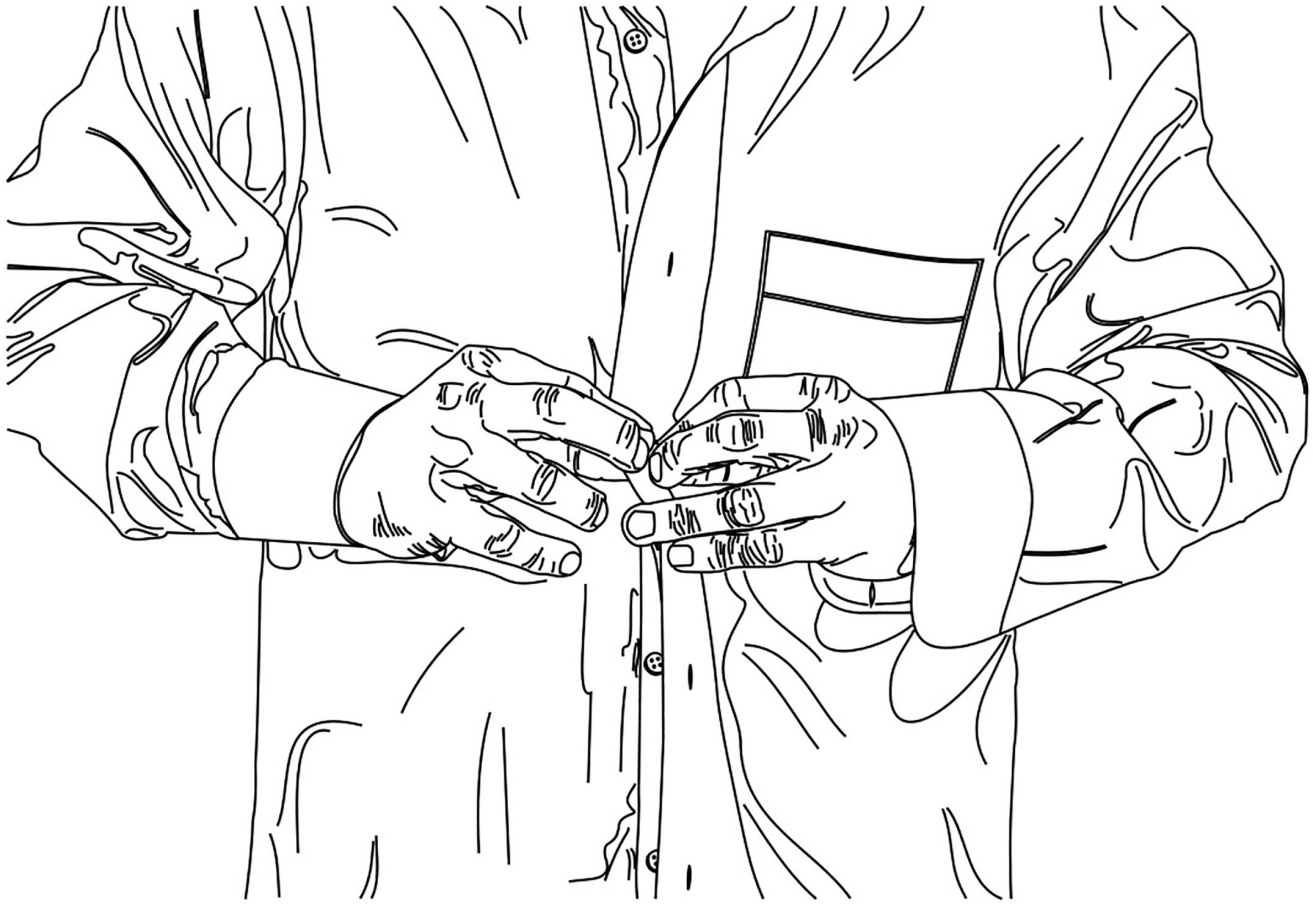
Functional performance. Shirt task. The standing participant picked up a folded unbuttoned long sleeve shirt and put it on as fast as possible. The test was completed when all six buttons were done-up in their corresponding holes.

Shirt task. The shirt task was adapted from the t-shirt test used in spinal cord injury research [27,28]. The standing participant was instructed to pick up a folded unbuttoned long sleeve shirt placed on a table directly in front of them and put it on as fast as possible (Fig 8). The test was completed when all six buttons (not including the collar and sleeve buttons) were done-up in their corresponding holes. The sex of the participant determined whether a male or female shirt was used (as the buttons and holes are on opposite sides for each gender). The time taken to complete the task (seconds) was recorded as the participant’s test score.

### Data and statistical analysis

Normative data values are presented as medians with 10^th^ and 90^th^ percentiles, categorised into the following age groups; 20–39, 40–59, 60–69,70–79, and 80 years and over (the younger groups were grouped within two age-groups: 20–39 and 40–59 as participants within these age-groups performed similarly). Due to a small proportion of missing data, all non-missing observations were used in the subsequent analyses. No data were imputed for these missing values.

All data were explored for normality prior to analysis. Variables with right-skewed distributions were transformed to their log_10_. For the reliability analysis, ICC (2,1) estimates for each test and their 95% confidence intervals were based on a single-rater, absolute agreement, 2-way random-effects model. The benchmarks suggested by Altman [29] were used to interpret the ICC scores (0.81–1.00 excellent reliability, 0.61–0.80 good reliability, 0.41–0.60 moderate reliability, 0.21–0.40 fair reliability, and <0.20 poor reliability). Both the coefficients of variation (CV) of measurement error and 95% limits of agreement were calculated to determine the absolute trial variability in scores for each test. Each parameter was calculated using the methods described by Portney & Watkins [30] and Bland & Altman [31] respectively.

Independent t-tests were used for group comparisons. Correlations between test performance, age and the shirt task were assessed using Pearson correlations and a multiple regression analysis was performed with the shirt task—a global measure of upper extremity function—entered as the dependent variable and the remaining upper limb PPA test measures as independent (or ‘predictor’) variables. Initially, PPA test measures with univariate correlations with shirt test times <0.01 were entered using the stepwise procedure. Then in subsequent steps, age and gender were entered to determine if they could account for additional variance in shirts test beyond the explanatory upper limb PPA test measures. Finally, a principal component analysis was conducted with oblique rotation (direct oblimin) on the upper limb PPA test measures. This analysis excluded the functional shirt test and included only two of the arm stability measures (as these measures were highly correlated). Sampling adequacy for the analysis was examined with the Kaiser-Meyer-Olkin (KMO) measure and by determining a mean KMO value for all outcome measures [32]. All statistical analyses were completed using SPSS version 25.0.

## Results

### Participant characteristics

Table 1 shows demographic, anthropometric, contrast sensitivity, visual acuity, and self-reported upper limb function measures for each age group and both genders. DASH scores indicated no limitations for those aged 20–59 [13,33]. In those aged 60+ years, the prevalence of reported difficulties performing normal daily activities increased with age. The visual acuity and contrast sensitivity scores indicated all participants had adequate vision to complete the upper limb tasks.

**Table 1:** Participant characteristics (mean±SD).

					**Males**				
**Age group**	**n**	**Age**	**Height (cm)**	**Weight (kg)**	**Body mass index**	**Handedness (right) (%)**	**Contrast sensitivity (MET)**	**Visual acuity (logMAR)**	**DASH**
**20–29**	37	26.1 (2.4)	179.8 (7.0)	80.9 (14.2)	24.9 (3.7)	32 (86%)	23.6 (0.8)	-0.14 (0.12)	1.6 (3.3)
**30–39**	33	34.3 (3.0)	178.2 (7.4)	78.5 (12.7)	24.7 (3.3)	31 (94%)	23.4 (0.9)	-0.11 (0.10)	1.7 (2.5)
**40–49**	20	44.4 (2.8)	176.0 (7.4)	77.1 (9.9)	24.9 (2.6)	20 (100%)	23.4 (1.1)	0.00 (0.18)	1.3 (2.6)
**50–59**	21	55.6 (2.4)	177.4 (6.2)	80.2 (10.5)	25.4 (2.6)	20 (95%)	22.7 (1.2)	0.13 (0.21)	1.3 (1.8)
**60–69**	20	65.3 (3.4)	178.4 (7.5)	81.3 (12.0)	24.7 (4.9)	16 (80%)	22.5 (1.7)	0.14 (0.22)	3.0 (4.3)
**70–79**	21	73.3 (1.9)	177.3 (6.9)	83.2 (12.5)	26.5 (3.6)	20 (95%)	22.0 (1.8)	0.13 (0.19)	4.3 (6.7)
**≥80**	20	86.4 (2.8)	173.9 (5.6)	76.3 (9.4)	25.2 (2.9)	19 (95%)	21.0 (1.9)	0.15 (0.13)	10.0 (9.1)
**Total**	172	50.7 (20.8)	177.6 (7.0)	79.7 (12.0)	25.1 (3.4)	158 (92%)	22.8 (1.6)	0.01 (0.20)	3.0 (5.3)
					**Females**				
**Age group**	**n**	**Age**	**Height (cm)**	**Weight (kg)**	**Body mass index**	**Handedness (right) (%)**	**Contrast sensitivity (MET)**	**Visual acuity (logMAR)**	**DASH**
**20–29**	40	24.9 (2.3)	164.8 (6.6)	63.9 (13.4)	23.4 (4.0)	35 (88%)	23.7 (0.8)	-0.12 (0.08)	2.1 (2.7)
**30–39**	37	33.4 (2.7)	166.1 (8.3)	62.5 (10.2)	22.3 (3.8)	34 (92%)	23.9 (0.2)	-0.11 (0.09)	3.0 (3.4)
**40–49**	24	44.5 (2.6)	165.4 (6.6)	67.0 (9.6)	24.5 (3.2)	22 (92%)	23.4 (1.2)	-0.02 (0.19)	3.4 (3.5)
**50–59**	20	55.5 (3.1)	164.4 (5.7)	70.3 (16.3)	26.1 (6.3)	18 (90%)	22.9 (1.7)	0.15 (0.26)	6.3 (7.9)
**60–69**	28	66.6 (2.3)	163.0 (7.5)	69.2 (14.0)	25.6 (6.3)	27 (96%)	23.0 (1.0)	0.03 (0.14)	7.8 (7.9)
**70–79**	25	74.0 (2.4)	161.3 (5.9)	69.3 (11.9)	26.7 (4.9)	23 (92%)	22.5 (1.5)	0.06 (0.14)	10.6 (10.9)
**≥80**	21	84.2 (3.6)	160.5 (6.2)	61.2 (9.8)	23.8 (3.4)	18 (86%)	21.4 (2.4)	0.15 (0.15)	17.0 (14.0)
**Total**	195	50.8 (20.9)	163.9 (7.0)	65.9 (12.6)	24.4 (4.8)	177 (91%)	23.1 (1.5)	-0.01 (0.18)	6.4 (8.8)

Note: MET scored on a scale of 1 to 24, with higher scores indicating better performance. logMAR scored on a -0.30 to 2.00 scale, with lower scores indicating better performance.

### Normative values

**Fig 9:**
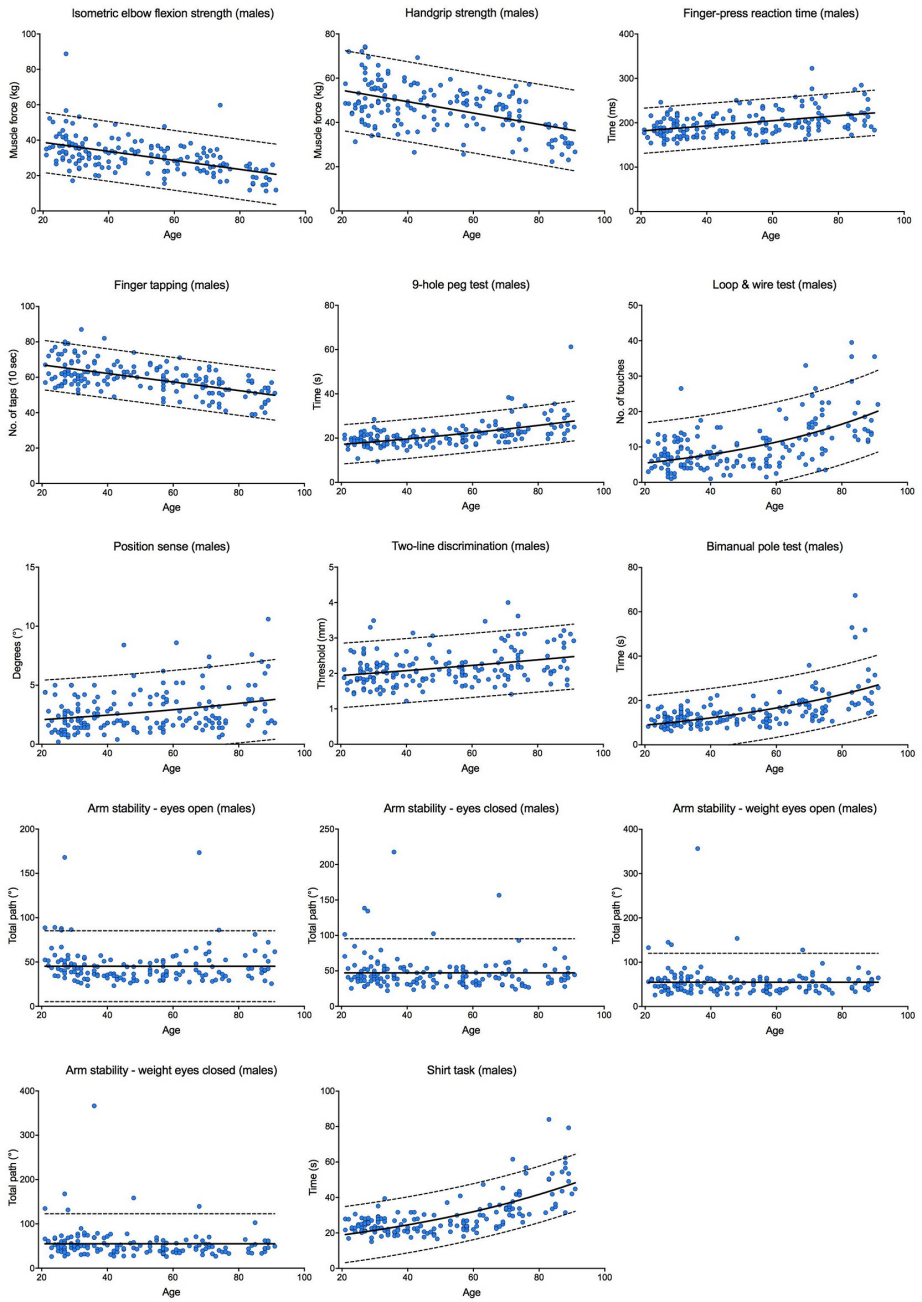
Individual test performance plotted against age in males. Individual participant scores are plotted against age for each of the continuously scored tests. The continuous black line represents the regression line and the broken black lines represent the 95% prediction bands.

**Fig 10:**
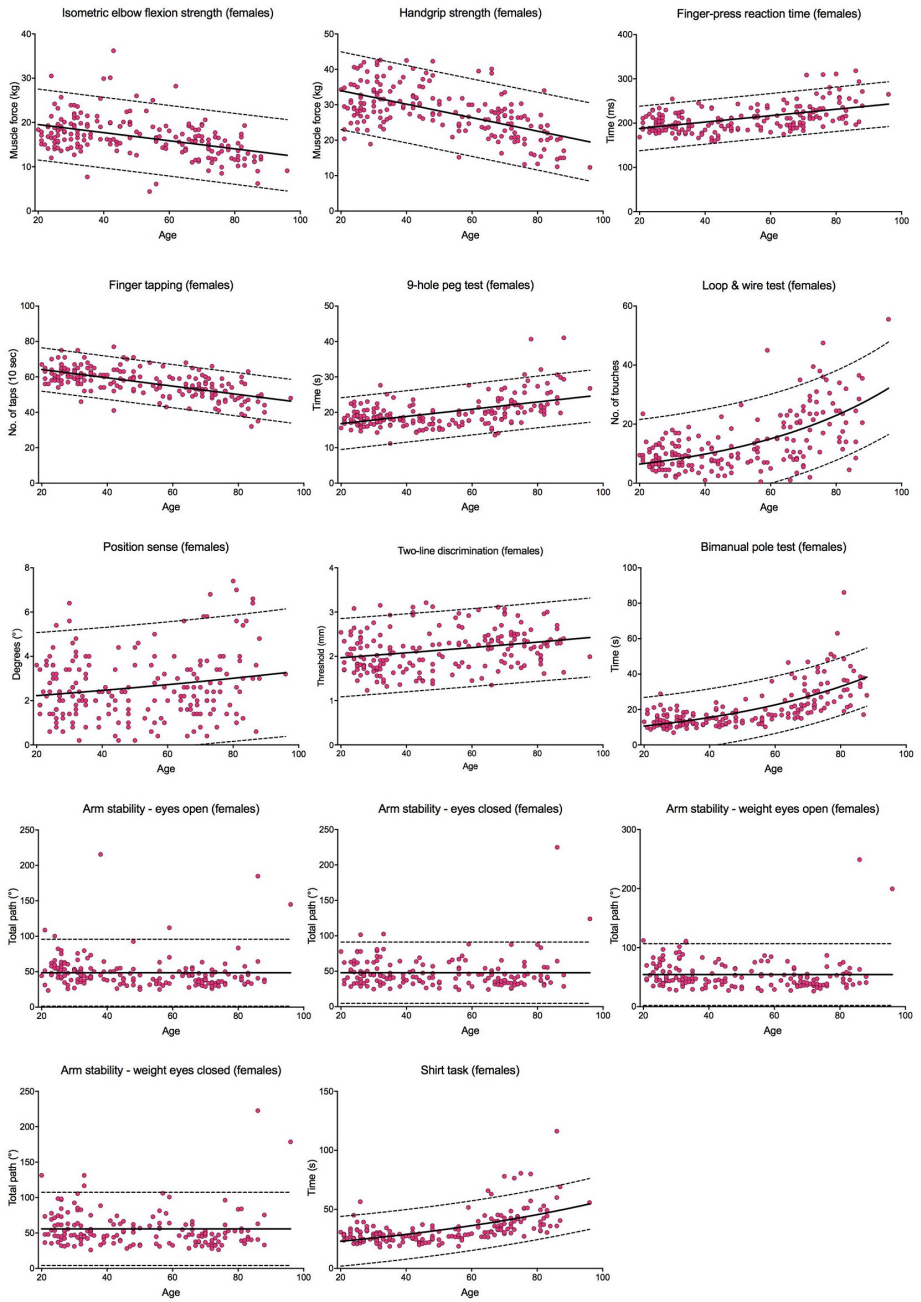
Individual test performance plotted against age in females. Individual participant scores are plotted against age for each of the continuously scored tests. The continuous black line represents the regression line and the broken black lines represent the 95% prediction bands.

Tables 2 and 3 report the median scores, the interquartile ranges and the 10^th^ and 90^th^ percentiles for each test within the upper limb PPA in each age group for males and females, respectively. Scores for the continuously scored tests are plotted against age in Figs 9 and 10; each graph fitted with a regression line and 95% prediction bands.

**Table 2:** Reference values (percentiles) for males in each upper limb PPA test for age groups 20–39, 40–49, 50–59, 60–69, 70–79 and ≥80.

Test	Age group	*n*	Missing data	10%	25%	50%	75%	90%
Isometric elbow flexion strength (kg)	20–39	69	1	24.5	28.4	33.7	39.7	49.5
	40–59	41	-	21.8	27.4	33.1	35.7	41.1
	60–69	20	-	22.6	24.6	28.8	32.6	36.8
	70–79	20	1	17.1	21.9	25.8	31.1	34.7
	≥80	20	-	11.8	15.0	18.3	23.4	26.0
Handgrip strength (kg)	20–39	70	-	38.2	44.1	49.3	58.2	65.7
	40–59	41	-	36.0	42.6	49.1	53.1	57.4
	60–69	20	-	38.9	42.9	47.2	50.8	54.2
	70–79	21	-	28.6	38.3	42.8	50.9	55.9
	≥80	20	-	23.3	28.9	31.3	37.7	39.2
Finger-press reaction time (ms)	20–39	70	-	163.4	169.1	183.6	200.6	217.9
	40–59	41	-	168.5	179.6	197.8	215.1	236.4
	60–69	20	-	180.8	190.4	201.6	230.2	248.0
	70–79	21	-	175.1	190.7	208.0	238.9	273.0
	≥80	20	-	183.9	191.8	213.0	228.8	273.8
Finger tapping (no. of taps)	20–39	70	-	55.1	58.0	62.0	69.3	75.0
	40–59	41	-	53.2	58.0	61.0	64.5	67.8
	60–69	20	-	46.3	54.3	58.0	61.0	65.0
	70–79	21	-	43.2	46.0	53.0	57.5	64.0
	≥80	20	-	39.1	45.3	51.5	56.3	58.0
9-hole peg test (sec)	20–39	70	-	15.7	17.1	18.7	20.8	22.8
	40–59	41	-	16.4	18.3	19.6	21.6	23.7
	60–69	20	-	17.6	20.5	22.2	24.0	27.4
	70–79	21	-	19.4	21.4	22.8	26.1	37.2
	≥80	20	-	19.9	22.4	25.8	30.9	35.2
Loop & wire test (no. of touches)	20–39	70	-	3.1	4.0	7.0	10.3	13.0
	40–59	41	-	2.4	4.5	6.5	10.3	12.5
	60–69	20	-	4.6	7.0	10.8	15.8	21.9
	70–79	21	-	4.1	9.3	15.5	19.8	24.1
	≥80	19	1	12.0	13.5	16.0	22.5	35.5
Position sense (°)	20–39	70	-	0.8	1.2	2.0	2.8	4.2
	40–59	41	-	1.0	1.6	2.2	4.0	5.1
	60–69	20	-	1.4	1.6	2.4	4.5	5.7
	70–79	21	-	0.9	1.6	2.0	3.2	6.2
	≥80	20	-	1.6	2.0	3.6	5.8	7.5
Tactile sensitivity (g)	20–39	70	-	0.02	0.02	0.04	0.04	0.07
	40–59	41	-	0.02	0.04	0.07	0.16	0.35
	60–69	20	-	0.02	0.04	0.07	0.16	0.40
	70–79	21	-	0.04	0.04	0.07	0.16	0.40
	≥80	20	-	0.04	0.07	0.16	0.40	0.40
Two-point discrimination (mm)	20–39	70	-	2.0	2.0	3.0	3.0	3.0
	40–59	41	-	2.0	2.0	3.0	3.0	4.0
	60–69	20	-	2.1	3.0	3.0	4.0	4.0
	70–79	21	-	2.0	3.0	4.0	5.0	6.0
	≥80	20	-	2.0	3.0	3.5	5.0	5.0
Two-line discrimination (mm)	20–39	70	-	1.5	1.8	2.0	2.2	2.6
	40–59	41	-	1.7	1.8	2.0	2.3	2.6
	60–69	20	-	1.6	1.8	2.1	2.3	3.0
	70–79	21	-	1.8	2.0	2.4	3.0	3.5
	≥80	20	-	1.7	2.1	2.3	2.9	3.1
Bimanual pole test (sec)	20–39	69	1	7.8	9.1	11.3	13.8	17.1
	40–59	41	-	8.3	10.0	11.8	14.3	18.0
	60–69	20	-	9.8	14.9	17.0	19.5	20.2
	70–79	21	-	10.8	12.6	15.4	24.7	27.7
	≥80	20	-	15.1	18.6	23.7	33.3	52.8
Arm stability—Total path eyes open (°)	20–39	63	7	30.8	35.7	43.4	52.4	78.3
	40–59	40	1	28.6	30.0	35.8	41.4	49.6
	60–69	18	2	29.0	31.6	38.8	57.9	76.6
	70–79	14	7	28.9	33.3	39.8	51.9	78.7
	≥80	17	3	28.1	36.1	42.4	59.5	74.0
Arm stability—Total path eyes closed (°)	20–39	63	7	31.5	35.6	43.2	56.3	75.1
	40–59	40	1	27.9	30.7	36.4	42.5	51.3
	60–69	18	2	29.5	31.2	39.9	53.9	77.9
	70–79	14	7	26.1	28.9	40.6	50.4	72.5
	≥80	16	4	31.0	36.5	41.5	56.8	72.5
Arm stability—Total path weight eyes open (°)	20–39	63	7	35.1	42.2	50.0	62.7	85.5
	40–59	40	1	29.2	32.0	38.8	47.6	60.3
	60–69	18	2	30.7	36.6	45.3	60.2	77.2
	70–79	13	8	32.4	39.5	46.5	58.8	89.4
	≥80	16	4	36.5	42.2	50.0	62.2	79.8
Arm stability—Total path weight eyes closed (°)	20–39	63	7	36.5	45.6	50.6	68.7	82.8
	40–59	40	1	28.3	33.4	39.0	48.4	58.1
	60–69	18	2	29.7	38.1	44.2	56.4	71.5
	70–79	12	9	28.5	36.8	44.8	47.4	52.3
	≥80	15	5	33.7	37.4	53.1	62.2	83.2
Shirt task (sec)	20–39	70	-	18.9	21.2	23.0	26.7	28.4
	40–59	41	-	18.1	20.7	24.0	26.9	31.4
	60–69	20	-	22.0	23.7	28.9	34.0	44.3
	70–79	21	-	29.5	30.7	34.9	42.1	56.2
	≥80	20	-	31.7	37.7	49.6	56.0	77.6

**Table 3:** Reference values (percentiles) for females in each upper limb PPA test for age groups 20–39, 40–49, 50–59, 60–69, 70–79 and ≥80.

Test	Age group	*n*	Missing data	10%	25%	50%	75%	90%
Isometric elbow flexion strength (kg)	20–39	77	-	14.1	15.7	17.9	20.0	23.3
	40–59	44	-	13.4	15.4	17.1	20.0	25.5
	60–69	28	-	11.5	13.8	16.1	18.7	19.6
	70–79	25	-	9.6	11.9	14.0	16.0	17.2
	≥80	21	-	9.0	10.7	11.8	14.1	17.3
Handgrip strength (kg)	20–39	77	-	24.6	28.1	30.6	35.7	40.3
	40–59	43	1	23.2	26.1	30.0	32.3	38.7
	60–69	28	-	20.3	22.9	26.6	28.7	39.0
	70–79	25	-	16.0	20.1	24.0	26.6	30.2
	≥80	21	-	12.7	15.0	20.1	23.1	26.5
Finger-press reaction time (ms)	20–39	77	-	176.6	183.8	194.9	211.9	226.2
	40–59	44	-	169.3	184.8	197.6	214.9	234.7
	60–69	28	-	183.0	193.5	201.5	225.4	246.1
	70–79	25	-	199.9	211.7	225.9	247.7	291.9
	≥80	21	-	200.7	212.2	246.3	268.3	307.7
Finger tapping (no. of taps)	20–39	77	-	56.0	58.0	61.0	64.5	67.2
	40–59	44	-	48.5	53.3	58.5	63.0	69.0
	60–69	28	-	49.0	52.0	54.0	59.0	64.1
	70–79	25	-	43.8	47.0	52.0	56.0	59.4
	≥80	21	-	35.6	41.0	47.0	49.5	59.4
9-hole peg test (sec)	20–39	77	-	15.9	17.0	18.2	19.3	22.5
	40–59	43	1	15.0	16.4	17.6	19.5	21.3
	60–69	28	-	15.0	17.9	20.0	23.2	26.4
	70–79	25	-	17.3	19.9	22.1	24.5	29.6
	≥80	21	-	19.9	21.4	24.2	28.8	31.8
Loop & wire test (no. of touches)	20–39	77	-	4.4	6.0	9.0	12.0	15.2
	40–59	44	-	3.0	6.5	8.5	12.4	20.0
	60–69	28	-	5.8	8.6	15.0	20.9	24.5
	70–79	25	-	8.5	17.5	24.0	30.8	38.6
	≥80	21	-	9.1	14.0	23.5	30.3	36.3
Position sense (°)	20–39	77	-	1.0	1.6	2.4	3.5	4.0
	40–59	44	-	0.5	1.2	2.1	2.8	4.2
	60–69	28	-	0.8	1.6	2.2	3.0	3.8
	70–79	25	-	1.2	1.9	2.6	3.7	5.8
	≥80	21	-	1.0	2.5	3.6	5.6	6.9
Tactile sensitivity (g)	20–39	77	-	0.01	0.01	0.02	0.04	0.05
	40–59	43	1	0.01	0.02	0.04	0.07	0.07
	60–69	28	-	0.02	0.03	0.04	0.06	0.18
	70–79	25	-	0.02	0.04	0.07	0.07	0.11
	≥80	20	1	0.03	0.07	0.16	0.40	0.58
Two-point discrimination (mm)	20–39	77	-	2.0	2.0	3.0	3.0	4.0
	40–59	44	-	2.0	2.0	3.0	3.0	4.0
	60–69	28	-	2.9	3.0	4.0	4.0	5.1
	70–79	25	-	2.0	3.0	3.0	4.0	5.4
	≥80	20	1	2.0	3.0	4.0	5.0	5.0
Two-line discrimination (mm)	20–39	76	1	1.5	1.7	1.9	2.2	2.6
	40–59	44	-	1.5	1.7	2.1	2.6	3.0
	60–69	26	2	1.6	2.2	2.4	2.8	3.1
	70–79	25	-	1.8	2.0	2.3	2.5	2.7
	≥80	21	-	1.7	2.0	2.3	2.4	2.9
Bimanual pole test (sec)	20–39	77	-	9.8	11.3	12.8	16.3	19.5
	40–59	43	1	10.9	12.6	15.0	18.0	23.0
	60–69	27	1	15.4	17.8	22.0	28.6	38.4
	70–79	25	-	19.0	22.3	29.8	42.6	50.5
	≥80	19	2	19.0	27.2	32.9	38.5	48.5
Arm stability—Total path eyes open (°)	20–39	70	7	34.8	41.5	50.2	60.8	75.8
	40–59	38	6	27.8	32.4	38.4	50.2	64.0
	60–69	23	5	30.5	35.8	44.4	48.7	55.3
	70–79	21	4	29.0	31.3	35.1	45.8	52.3
	≥80	18	3	32.6	35.8	42.7	59.2	148.9
Arm stability—Total path eyes closed (°)	20–39	70	7	34.6	41.1	51.0	64.5	78.0
	40–59	39	5	27.8	31.0	39.4	45.8	67.1
	60–69	23	5	30.7	33.8	40.3	46.2	53.9
	70–79	20	5	26.8	31.9	38.6	45.3	62.3
	≥80	17	4	29.7	35.4	44.7	60.1	144.2
Arm stability—Total path weight eyes open (°)	20–39	70	7	40.2	47.9	62.0	77.5	92.4
	40–59	39	5	30.0	35.1	42.4	54.7	75.2
	60–69	22	6	34.2	37.0	44.2	53.9	65.6
	70–79	20	5	30.8	35.2	38.7	49.0	66.5
	≥80	18	3	36.7	40.7	54.6	64.8	204.6
Arm stability—Total path weight eyes closed (°)	20–39	70	7	40.6	49.6	62.6	77.7	98.5
	40–59	39	5	31.6	35.4	44.1	60.1	81.4
	60–69	21	7	31.6	37.6	47.7	53.8	61.9
	70–79	20	5	28.3	32.7	43.1	49.9	60.6
	≥80	17	4	34.6	40.6	52.2	69.7	187.5
Shirt task (sec)	20–39	77	-	21.0	23.8	27.0	30.2	33.8
	40–59	43	1	21.0	24.4	26.8	29.4	34.8
	60–69	28	-	26.8	30.6	35.5	41.6	47.1
	70–79	25	-	30.2	34.5	42.4	51.2	78.8
	≥80	18	3	31.6	35.9	41.5	53.7	73.7

Note: low scores in isometric elbow flexion strength, handgrip strength and finger tapping indicate worse performance. Low scores in all other tests indicate better performance.

**Fig 11:**
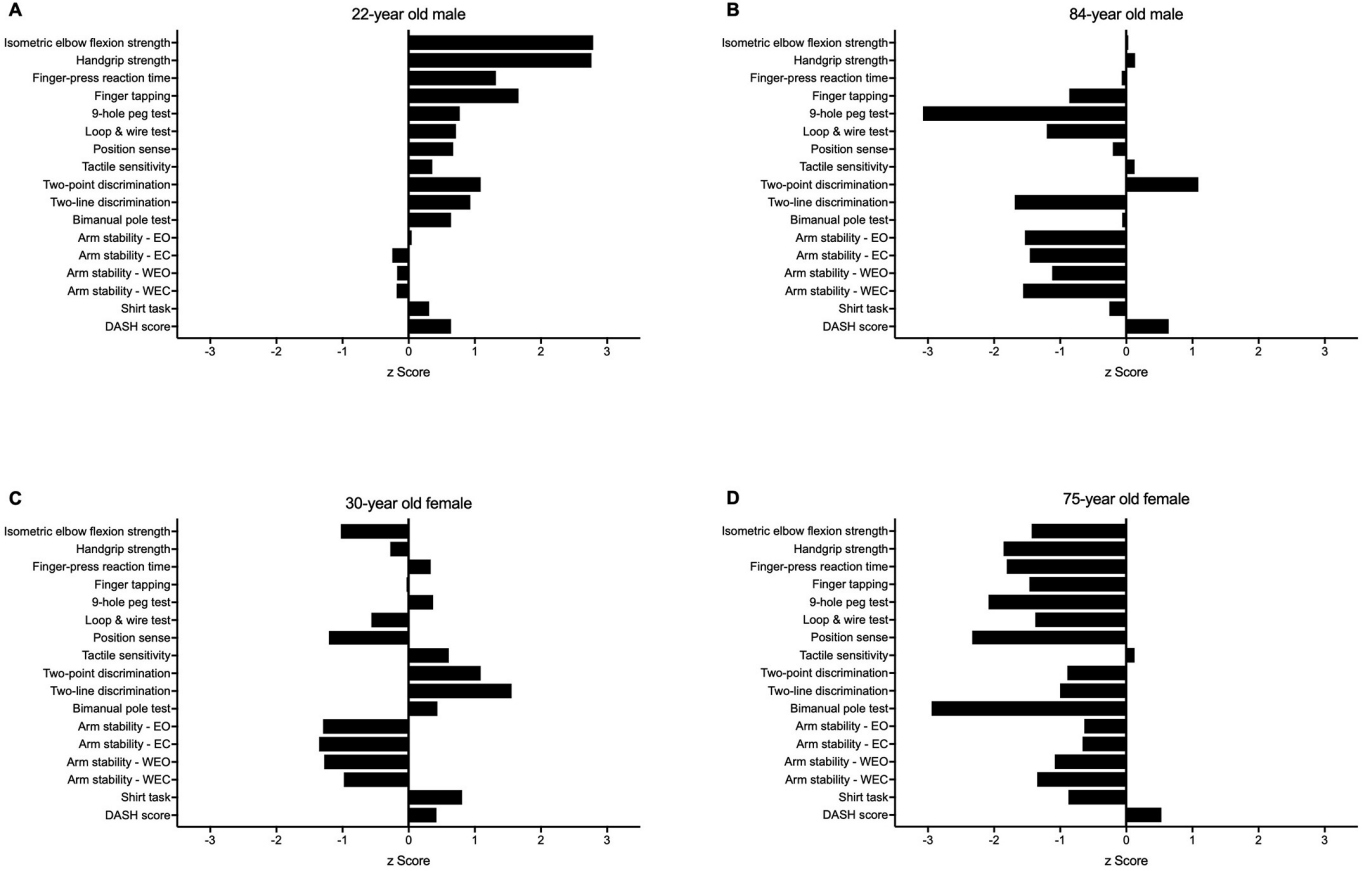
Upper limb Physiological Profile Assessment (PPA) z-score output for four participants. Test scores are presented as standardised (z) scores, referenced to the entire sample, to allow direct comparison in performance between each test both within and between individuals. Each unit represents one standard deviation. A score of zero indicates an average level of performance compared to the study population, while positive and negative scores represent above- and below-average performances respectively. **(A)** A 22-year old male who, in general, performed well above average in the majority of tests. **(B)** An 84-year old male with below average levels of coordination and arm stability, while maintaining average levels of muscle strength. **(C)** A 30-year old female demonstrating above average skin sensibility, but below average levels of muscle strength and arm stability. **(D)** A 75-year old female who performed below average in most tests.

Fig 11 provides examples of individual performance profiles for four participants.

### Test-retest reliability

Test-retest statistics for each test are shown in Table 4. Intra-class correlation coefficients (ICC) for isometric elbow flexion strength, handgrip strength, finger-press reaction time, finger tapping, bimanual pole test, and both weighted conditions of the arm stability test were excellent (ranging from 0.81 to 0.98). Good reliability was attained the 9-hole peg test, loop and wire test, tactile sensitivity, two-point discrimination, two-line discrimination, both unweighted conditions of the arm stability test, and the shirt task (ranging from 0.65 to 0.79). Position sense only attained a fair level of test-retest reliability (0.31).

**Table 4:** Intra-class correlation coefficients, coefficients of variation and 95% limits of agreement for each test.

Measure	ICC [95% CI]	CV (%)	Mean [95%LoA]
Isometric elbow flexion strength (kg)	.95 [.89, .97]	7.9	23.9 [17.9, 28.5]
Handgrip strength (kg)	.98 [.96, .99]	5.1	40.2 [34.1, 45.6]
Finger-press reaction time (ms)	.81 [.63, .90]^a^	7.2	202.5 [167.6, 247.1]
Finger tapping (no. of taps)	.83 [.66, .92]	4.9	59.9 [50.7, 66.1]
9-hole peg test (s)	.75 [.54, .87]^a^	10.3	20.2 [15.4, 26.7]
Loop & wire test (no. of touches)	.75 [.54, .88]^a^	34.0	13.2 [1.2, 26.3]
Position sense (°)	.31 [-.06, .60]^a^	40.9	3.2 [-2.2, 8.2]
Tactile sensitivity (g)	.76 [.56, .88]^a^	55.0	0.1 [0.0, 0.3]
Two-point discrimination (mm)	.71 [.47, .85]^a^	15.2	2.8 [1.6, 4.1]
Two-line discrimination (mm)	.65 [.39, .82]	8.9	2.0 [1.5, 2.5]
Bimanual pole test (s)	.87 [.68, .94]^a^	18.2	16.6 [9.9, 26.1]
Arm stability—Total path eyes open^b^ (°)	.76 [.54, .88]^a^	14.9	46.5 [24.9, 62.0]
Arm stability—Total path eyes closed^b^ (°)	.76 [.55, .88]^a^	13.0	45.6 [27.7, 60.6]
Arm stability—Total path weight eyes open^b^ (°)	.81 [.64, .91]^a^	13.1	53.2 [31.9, 70.5]
Arm stability—Total path weight eyes closed^b^ (°)	.82 [.65, .91]^a^	14.0	55.2 [32.3, 75.5]
Shirt task (s)	.75 [.53, .87]^a^	14.5	31.5 [18.5, 44.3]

^a^log_10_ transformed prior to statistical testing due to skewed data

^b^missing data from one participant (n = 29)

ICC, intra-class correlation coefficient; CV, coefficient of variation; LoA, limits of agreement

CVs were small (<20%) for isometric elbow flexion strength, handgrip strength, finger-press reaction time, finger tapping, 9-hole peg test, two-point discrimination, two-line discrimination, bimanual pole test, all arm stability test outcomes and the shirt task (4.9–18.2%), but relatively large for the three remaining tests (34.0%, 40.9% and 55.0% for the loop and wire, position sense and tactile sensitivity tests, respectively). This was consistent with the 95% limits of agreement.

### Gender differences and associations with ageing

Table 5 presents differences in mean scores between males and females for each test. Women performed better than men in the 9-hole peg test (*t* = 2.89, *p* = 0.004) and tactile sensitivity (*t* = 4.75, *p* < 0.001) tests. Men performed better than women in the remaining tests with the exception of position sense, two-point discrimination, two-line discrimination and the eyes open unweighted arm stability test condition where there were no gender differences.

**Table 5:** Mean (±SD) scores for each gender for each test, difference in performance between genders (independent t-tests).

Measure	Males (n = 172)	Females (n = 195)	Difference [95% CI]	Independent t-tests
	Mean (SD)	Mean (SD)		
Isometric elbow flexion strength^a^ (kg)	31.0 (10.0)	16.7 (4.4)	14.2 [12.6, 15.9]	<.001
Handgrip strength (kg)	46.6 (10.5)	28.1 (6.8)	18.5 [16.7, 20.4]	<.001
Finger-press reaction time^a^ (ms)	199.2 (28.1)	210.2 (29.4)	-11.0 [-16.9, -5.1]	<.001
Finger tapping (no. of taps)	59.6 (8.6)	57.0 (7.9)	2.6 [0.9, 4.3]	.002
9-hole peg test^a^ (s)	21.3 (5.4)	19.9 (4.2)	1.4 [0.4, 2.4]	.004
Loop & wire test^a^ (no. of touches)	10.3 (6.9)	13.7 (9.6)	-3.4 [-5.1, -1.7]	.001
Position sense^a^ (°)	2.7 (1.7)	2.6 (1.5)	0.1 [-0.2, 0.5]	.529
Tactile sensitivity^a^ (g)	0.10 (0.15)	0.07 (0.10)	0.04 [0.01, 0.07]	<.001
Two-point discrimination (mm)	3.1 (1.0)	3.1 (1.0)	-0.1 [-0.3, 0.2]	.611
Two-line discrimination (mm)	2.2 (0.5)	2.1 (0.5)	0.0 [-0.1, 0.1]	.718
Bimanual pole test^a^ (s)	15.3 (8.3)	20.4 (11.3)	-5.1 [-7.1, -3.0]	<.001
Arm stability—Total path eyes open^a^ (°)	45.1 (20.2)	48.8 (23.8)	-3.7 [-8.5, 1.2]	.069
Arm stability—Total path eyes closed^a^ (°)	45.1 (18.9)	49.9 (26.1)	-4.7 [-9.8, 0.3]	.047
Arm stability—Total path weight eyes open^a^ (°)	50.9 (19.6)	58.0 (36.0)	-7.1 [-13.4, -0.8]	.035
Arm stability—Total path weight eyes closed^a^ (°)	51.3 (21.2)	59.4 (35.9)	-8.1 [-14.5, -1.6]	.011
Shirt task^a^ (s)	29.5 (11.3)	33.4 (13.1)	-3.9 [-6.4, -1.3]	<.001

^a^log_10_ transformed prior to statistical testing due to skewed data

Note: low scores in isometric elbow flexion strength, handgrip strength and finger tapping indicate worse performance. Low scores in all other tests indicate better performance.

With the exception of arm stability, performance in all tests decreased with age (Table 6). These correlations were considered strong (–1.0 to –0.5 or 0.5 to 1.0) for finger tapping (*r* = –0.60, *p* < 0.001), 9-hole peg test (*r* = 0.53, *p* < 0.001), tactile sensitivity (*r* = 0.60, *p* < 0.001), bimanual pole test (*r* = 0.64, *p* < 0.001), and the shirt task (*r* = 0.64, *p* < 0.001). Correlations between performance and age were considered moderate (–0.5 to –0.3 or 0.3 to 0.5) for all other tests, except for position sense (*r* = 0.18, *p* = 0.001) and two-line discrimination (*r* = 0.29, *p* < 0.001), which were considered as weak (0.1 to 0.3). Weak but significant associations for better arm stability and age were evident for all four test conditions (*r* = –0.22 to –0.12, *p* = <0.001 to 0.034).

**Table 6:** Correlations between individual test performance and age.

Measure	Mean (SD)	Age (*r*)	*p*
Isometric elbow flexion strength^a^ (kg)	23.4 (10.4)	-0.36	<.001
Handgrip strength (kg)	36.8 (12.7)	-0.36	<.001
Finger-press reaction time^a^ (ms)	205.1 (29.3)	0.46	<.001
Finger tapping (no. of taps)	58.2 (8.3)	-0.60	<.001
9-hole peg test^a^ (s)	20.6 (4.8)	0.53	<.001
Loop & wire test^a^ (no. of touches)	12.1 (8.6)	0.48	<.001
Position sense^a^ (°)	2.7 (1.6)	0.18	.001
Tactile sensitivity^a^ (g)	0.09 (0.13)	0.60	<.001
Two-point discrimination (mm)	3.1 (1.0)	0.42	<.001
Two-line discrimination (mm)	2.2 (0.5)	0.29	<.001
Bimanual pole test^a^ (s)	18.0 (10.3)	0.64	<.001
Arm stability—Total path eyes open^a^ (°)	47.0 (22.2)	-0.12	.034
Arm stability—Total path eyes closed^a^ (°)	47.6 (23.0)	-0.16	.005
Arm stability—Total path weight eyes open^a^ (°)	54.7 (29.6)	-0.17	.003
Arm stability—Total path weight eyes closed^a^ (°)	55.6 (30.2)	-0.22	<.001
Shirt task^a^ (s)	31.5 (12.4)	0.64	<.001

^a^log_10_ transformed prior to statistical testing due to skewed data

Note: Negative *r* values for isometric elbow flexion strength, handgrip strength and finger tapping indicate worsening performance with increasing age. Positive *r* values for all other tests indicate worsening performance with increasing age.

### Test performance in those with and without self-reported upper-limb impairment

Participants were classified as having an upper-extremity impairment if they scored >15/100 on the DASH questionnaire [13]. Only two out of the 232 participants aged 20–59 years had DASH scores >15. In the participants aged 60 years and over (n = 135), those with impairment (n = 25) performed significantly worse than those without impairment (n = 107) in handgrip and elbow flexion strength, reaction time, finger tapping, loop and wire, tactile sensitivity and bimanual pole tests as well as in the composite shirt task (Table 7).

**Table 7:** Mean (±SD) scores for each DASH category (those who scored ≤15 vs. those who scored >15) for each test in those aged 60 years and over, difference in performance between DASH categories (independent t-tests).

Measure	n	DASH ≤15	DASH >15	n	Difference [95% CI]	Independent t-tests
		Mean (SD)	Mean (SD)			
Isometric elbow flexion strength^a^ (kg)	106	20.4 (8.3)	14.1 (3.5)	25	6.2 [4.1, 8.4]	<.001
Handgrip strength (kg)	107	33.7 (11.5)	21.4 (5.5)	25	12.3 [9.2, 15.4]	<.001
Finger-press reaction time (ms)	107	216.6 (29.1)	242.4 (39.5)	25	-25.8 [-43.0, -8.7]	.004
Finger tapping (no. of taps)	107	53.6 (7.1)	48.4 (6.1)	25	5.2 [2.1, 8.2]	.001
9-hole peg test^a^ (s)	107	23.3 (6.1)	25.3 (5.6)	25	-2.0 [-4.7, 0.6]	.074
Loop & wire test (no. of touches)	107	17.5 (9.7)	22.5 (10.2)	25	-4.9 [-9.3, -0.5]	.028
Position sense^a^ (°)	107	2.9 (1.7)	3.6 (2.5)	25	-0.6 [-1.7, 0.4]	.314
Tactile sensitivity^a^ (g)	106	0.13 (0.18)	0.20 (0.18)	25	-0.07 [-0.15, 0.01]	.018
Two-point discrimination (mm)	106	3.6 (1.1)	3.8 (1.1)	25	-0.2 [-0.7, 0.3]	.379
Two-line discrimination (mm)	105	2.4 (0.5)	2.3 (0.5)	25	0.1 [-0.1, 0.3]	.412
Bimanual pole test^a^ (s)	105	24.4 (12.0)	33.5 (13.4)	24	-9.1 [-14.6, -3.6]	.001
Arm stability—Total path eyes open^a^ (°)	89	46.1 (26.0)	47.0 (12.4)	19	-0.9 [-13.0, 11.3]	.396
Arm stability—Total path eyes closed^a^ (°)	87	46.4 (27.9)	46.7 (14.0)	18	-0.3 [-13.7, 13.1]	.504
Arm stability—Total path weight eyes open^a^ (°)	85	52.5 (31.8)	53.2 (11.8)	19	-0.8 [-15.5, 14.0]	.352
Arm stability—Total path weight eyes closed^a^ (°)	82	50.8 (29.4)	53.1 (13.5)	18	-2.3 [-16.4, 11.8]	.275
Shirt task^a^ (s)	107	39.0 (12.0)	53.1 (21.3)	22	-14.0 [-23.7, -4.4]	<.001

^a^log_10_ transformed prior to statistical testing due to skewed data

Note: low scores in isometric elbow flexion strength; handgrip strength and finger tapping indicate worse performance. Low scores in all other tests indicate better performance.

### Associations between the sensorimotor tests and the composite measure of upper-limb function

Correlations between performance in the initial 16 upper limb PPA test measures and performance in the shirt task are presented in Table 8. All test measures, with the exception of the arm stability tests, were significantly associated with shirt test times. These correlations were considered strong (–1.0 to –0.5 or 0.5 to 1.0) for the bimanual pole test (*r* = 0.60, *p* < 0.001), and moderate for the remaining tests (–0.5 to –0.3 or 0.3 to 0.5), except for position sense and two-line discrimination, which were considered weak (0.1 to 0.3). The multiple regression revealed the bimanual pole, loop and wire, 9-hole peg test, finger-press reaction time, tactile sensitivity, isometric elbow flexion strength and two-line discrimination tests were significant and independent predictors of performance in the shirt task, with an *R*^2^ value of 0.48 (*p* < 0.001) (Table 9). The inclusion of age in the subsequent step explained a further 3% of the variance in shirt test times (*p* < 0.001) and the addition of gender in the final step contributed a further 1% (*p* = 0.014). The final model explained 52% of the variance in the performance of the shirt task.

**Table 8:** Correlations between individual upper limb test performance and performance in the shirt task.

Measure	Shirt task^a^ (*r*)	*p*
Isometric elbow flexion strength^a^ (kg)	-0.41	<.001
Handgrip strength (kg)	-0.40	<.001
Finger-press reaction time^a^ (ms)	0.44	<.001
Finger tapping (no. of taps)	-0.46	<.001
9-hole peg test^a^ (s)	0.45	<.001
Loop & wire test^a^ (no. of touches)	0.51	<.001
Position sense^a^ (°)	0.14	.009
Tactile sensitivity^a^ (g)	0.36	<.001
Two-point discrimination (mm)	0.32	<.001
Two-line discrimination (mm)	0.28	<.001
Bimanual pole test^a^ (s)	0.60	<.001
Arm stability—Total path eyes open^a^ (°)	0.04	.473
Arm stability—Total path eyes closed^a^ (°)	0.02	.970
Arm stability—Total path weight eyes open^a^ (°)	0.02	.736
Arm stability—Total path weight eyes closed^a^ (°)	-0.03	.606

^a^log_10_ transformed prior to statistical testing due to skewed data

**Table 9:** Hierarchical multiple regression of performance in the shirt task^a^, showing standardised Beta weights, and *R*^2^ after entry of each successive block of variables into the model.

Model	*β*	*p*	*R*^2^
Bimanual pole test^a^ (s)	.31	<.001	
Loop & wire test^a^ (no. of touches)	.14	.004	
9-hole peg test^a^ (s)	.14	.004	
Finger-press reaction time^a^ (ms)	.10	.043	
Tactile sensitivity^a^ (g)	.12	.008	
Isometric elbow flexion strength^a^ (kg)	-.12	.015	
Two-line discrimination (mm)	.10	.023	0.48***
Age	.28	<.001	0.51***
Gender	.16	.014	0.52*

^a^log_10_ transformed prior to statistical testing due to skewed data

***p < 0.001,

**p < 0.01,

*p < 0.05

Note: *n* = 348

### Exploration for potential latent factors

The principal component analysis revealed four factors with eigenvalues over Kaiser’s criterion of 1, which in combination explained 66.7% of the variance. Table 10 shows the factor loadings after rotation. The factor loadings suggest factor 1 represents manual and gross motor skills, factor 2 represents arm stability, factor 3 represents sensation and fine motor control, and factor 4 represents tactile discrimination thresholds.

**Table 10:** Summary of exploratory factor analysis results for the upper limb PPA. Four factors with eigenvalues >1 were identified and were named ‘manual and gross motor skills,’ ‘arm stability,’ sensation and fine motor control,’ and ‘tactile discrimination thresholds’.

Measure	Manual & gross motor skills	Arm stability	Sensation & fine motor control	Tactile discrimination thresholds
**Isometric elbow flexion strength**^a^ **(kg)**	**-.98**	-.00	.17	.09
**Handgrip strength (kg)**	**-.97**	-.01	.19	.09
**Bimanual pole test**^a^ **(s)**	**.60**	-.09	.26	.23
**Loop & wire test**^a^ **(no. of touches)**	**.52**	.19	.18	.22
**Finger-press reaction time**^a^ **(ms)**	**.51**	.00	.23	.14
**Finger tapping (no. of taps)**	**-.47**	.17	**-.43**	-.08
**Arm stability—Total path eyes open**^a^ **(°)**	-.00	**.96**	.03	.01
**Arm stability—Total path weight eyes closed**^a^ **(°)**	.02	**.95**	-.03	-.06
**Tactile sensitivity**^a^ **(g)**	-.12	-.15	**.76**	.14
**Position sense**^a^ **(°)**	.09	.02	**.67**	-.38
**9-hole peg test**^a^ **(s)**	.00	.17	**.65**	.31
**Two-line discrimination (mm)**	-.03	-.00	-.05	**.83**
**Two-point discrimination (mm)**	.15	-.10	.04	**.63**
**Eigenvalues**	4.03	2.10	1.47	1.07
**% of variance**	31.02	16.16	11.28	8.25

^a^log_10_ transformed prior to statistical testing due to skewed data

Note: Factor loadings over 0.40 appear in bold; *n* = 303

## Discussion

Our upper limb Physiological Profile Assessment (PPA) encompassed measures of muscle strength, unilateral movement and dexterity, position sense, skin sensation, bimanual coordination and arm stability. As such, it quantifies sensorimotor performance that we hypothesised would be important for upper limb function and provides normative test scores for people across the adult lifespan. As evident in Fig 11, this approach revealed markedly different performance profiles among individuals that could not be ascertained from a single composite assessment. With the exception of position sense, each test demonstrated good-to-excellent test-retest reliability when assessed in healthy individuals across a broad age range. The majority of tests showed good external validity against ageing and functional performance, with eight tests showing strong criterion validity in differentiating those aged 60 years and over with and without upper-extremity problems. Seven tests were able to explain 48% of the variance in performance of a composite measure of upper limb function, with age and gender contributing a further 4% when added to the model in subsequent steps. Lastly, an exploratory principal factor analysis indicates the upper limb PPA tests may be cluster within four factors comprising gross motor skills, arm stability, fine motor control and tactile discrimination.

### Normative values, gender differences and associations with ageing

Some tests included in the upper limb PPA have been previously well validated. These include handgrip strength [16], finger-press reaction time [17], 9-hole peg test [19,20], tactile sensitivity [24], and two-point discrimination [34–37]. Isometric elbow flexion strength [38], finger tapping [18] and position sense [17,21] have also been routinely measured, but a lack of standardisation and differences in testing protocol limit direct comparisons with earlier research.

Our handgrip strength data are generally consistent with previous research [39–42]. Sella [43] reported slightly lower mean scores at each age group, and Gilbertson & Barber-Lomax [44] noted a larger decline in performance in their older participants than reported here. It is important to acknowledge that the current study reports median scores at each age group, and therefore is less sensitive to outliers, for example, poor scores from frail older individuals. Furthermore, both previous studies tested handgrip at multiple handle positions [43] and multiple grip types [44], opening the possibility of fatigue influencing their results.

Reaction times to visual stimuli have been consistently reported to range between 180–200 ms in young people [17,45,46] with reaction times progressively increasing with age until the sixth decade and then slowing appreciably [47–50]. In addition, variability in response time increases during the latter years. Our results are consistent with this past work as are our findings that men have quicker reaction times than women across all age groups [45,50–52].

A decline in performance in the 9-hole peg test with increasing age, along with women performing the test quicker than men, is consistent with both Grice et al. [53] and Wang et al. [54]. Interestingly, our scores for each age group were approximately two seconds slower than those previously reported. One difference between studies was the orientation of the pegboard—previous studies aligned it lengthwise such that the participant moved the pegs right to left into the dish rather than straight ahead. Furthermore, a practice trial was permitted in the former studies, opening the possibility of a learning effect enhancing performance.

While several studies have reported age-related data for skin sensation [24,55], considerable variability in the anatomical locations assessed makes comparisons difficult. Bowden & McNulty [24] reported an increase in skin sensibility thresholds of 0.66 g and 0.25 g for males and females, respectively, between the ages of 20 to 80 when applying von Frey filaments to the hypothenar eminence of the dominant hand. While the magnitude of the reported increase [24] is far greater than the 0.12 g and 0.14 g reported in the current study, they reported an interaction between age and sex at the hypothenar eminence—with thresholds higher in men only after the age of 60. This gender difference is consistent across both studies.

Previous studies have also reported two-point discrimination thresholds increase with age [56,57]. Direct comparison of reference values with the current findings are fraught due to differences in methodology, such as the orientation of the two-points when applied to the participant’s skin [56]. For example, Bowden & McNulty [24] computed a composite sensibility measure from thresholds at three sites on the hand (two on the palm of the hand, one at the tip of the middle index finger). They found women had lower thresholds than men, but that if only the distal phalanx of the middle finger was considered, no gender differences were apparent. Men and women also perform similarly in tests that have measured two-point discrimination with a specialised two-point discrimination aesthesiometer [58] and a 5 mm thick sheet of Dow high-density Styrofoam [59].

A recent study has shown that performance in tactile sensitivity is more related to peripheral factors, while spatial discrimination, as measured in the current study by the two-point and two-line discrimination tests, are more associated with central processes [55]. The same study also showed that the latter were more strongly related to ageing, suggesting that tests like the two-point discrimination test have a greater cognitive component compared to tests of tactile sensitivity. This is something to consider for future development of a short-form version of the upper limb PPA.

Kotte et al. [38] recently performed a systematic review of studies reporting normative data for isometric elbow flexion strength. This included 1880 healthy volunteers across 19 studies. Assuming a flexion-extension moment arm of 26.4 cm, based on average forearm length reported by Askew et al. [60], mean values of 76.7 Nm and 52.5 Nm were calculated for men and women respectively. Applying the same formula to the current study reveals similar values for men (78.7 Nm), but lower values for women (42.5 Nm). This latter discrepancy may reflect considerable variability in experimental designs across studies. The lack of a standardised measurement protocol; i.e. devices used, the positioning of the participant (i.e. gravity eliminated vs. gravity assisted), the number of trials performed—and whether the best or average score was analysed, are among the many differences across the studies. Nonetheless, our results support the main findings of the systematic review, i.e. an inverse relationship between strength and age, and with greater levels of strength exhibited by males.

Performances in the novel tests (loop and wire, two-line discrimination, bimanual pole test and the shirt task) all declined significantly with age, except for the arm stability test, with performance unexpectedly improving with age. The assessment of two-line discrimination is a variant of that used by Carlson et al. [61] who quantified ability to accurately detect the change from one to two lines underneath the tip of their index finger (see Carlson et al. [61], Instrument D). Although men in the two studies performed similarly (1.76 mm vs. 2.1 mm), women in the Carlson et al. [61] study performed notably better (1.32 mm vs. 2.1 mm), leading to a significant difference between genders in their study. Possible age differences may explain this difference, however, the age of the participants in the study of Carlson et al. [61] was not reported.

The arm stability tests were designed to be analogous to the measurement of postural sway used in the original PPA [10,17], both in the outcome measures used (assessing the total path travelled) and in the use of four separate conditions. However, unlike the original postural sway test, weak but significant associations for arm stability and age were evident for all four conditions. It is possible that the tests were not sufficiently difficult to reveal any functional impairment across the adult lifespan.

Men and women performed similarly in the position sense, two-point discrimination, two-line discrimination and the eyes open unweighted arm stability test condition. In the remaining tests, men performed better in the tests of grip and elbow flexion strength, finger-press reaction time, finger tapping, loop and wire, bimanual coordination, the remaining arm stability test conditions and the shirt task. In contrast, women performed better than men in position sense, tactile sensitivity and the 9-hole peg tests. These gender differences are generally consistent with available literature [24, 45,50–52,54,58–60].

### Test-retest reliability

All but the test of proprioception had good-to-excellent test-retest reliability based on ICC scores, coefficients of variation [30] and limits of agreement [31]. However, there are a few caveats. For the tactile sensitivity test, the ICC was good (0.79) but the CV was high (55%). This likely resulted from the tactile sensitivity test being scored on a discrete logarithmic scale, which can make the CV vulnerable to inflation. Examination of threshold disparities shows nine participants (30%) recorded the same score on both test occasions, 17 participants (56.7%) had a disparity of 1 filament and only 4 participants (13.3%) had a disparity of 2 filaments. This supports the excellent test-retest reliability score obtained with the ICC. Furthermore, the scores for the 4 participants who had a disparity of 2 filaments between test and retest were 0.07 g and 0.02 g, representing differences at the upper end of the scale and therefore overall small differences in applied force. The ICC and standard deviations for test scores for the loop and wire test (see Table 4) were large which suggests high within-subject variability. This could be due to the test’s conflicting goals of navigating one’s way through the wire course as ‘fast’ *and* as ‘accurately’ as possible. It is possible some participants may have aimed for speed at the initial test while forgoing speed in place of minimising contacts at retest. Lastly, although position sense attained only a fair level of test-retest reliability, this is consistent with previous measures of position sense used in the lower limb [17].

### Criterion validity

To determine the criterion validity of the upper limb PPA tests, performance was compared between those with and without an upper limb impairment as indicated by the DASH questionnaire [13,33] in those aged 60 years and over. These analyses revealed significant differences for the muscle strength, dexterity, skin sensation, bimanual coordination domains as well as for the composite upper limb functional measure. Future studies of performance in the upper limb PPA tests in clinical groups may provide further insight into the criterion validity of the individual tests.

Previous work has found the sensory and motor tests of the original PPA could explain substantial variance in relevant composite functional measures such as gait speed [62], chair rise [63] and stair climbing abilities [64,65]. With the exception of the tests of arm stability, performance in the sensory and motor tests correlated with performance in the shirt task. Seven tests explained 48% of the variance in the performance in this composite measure. The beta weights from the regression analyses indicated the bimanual pole test was the most important measure for explaining shirt task times with the remaining tests (loop and wire test, 9-hole peg test, finger-press reaction time, tactile sensitivity, isometric elbow flexion strength and two-line discrimination) making lower, but still significant, contributions. The inclusion of age and gender in subsequent steps contributed a significant additional 4% of explained variance in shirt test times indicating our explanatory upper limb PPA measures accounted for most, but not all, age and gender effects.

The lack of significant associations between the arm stability tests with self-reported upper limb impairment and the shirt task suggests that although reliable, these tests are not measuring arm stability in a functionally valid way and therefore have little clinical utility for neurologically healthy cohorts.

### Exploratory investigation into potential latent factors

The principal component analysis identified four factors in which the upper limb PPA tests could be categorised. Both tests of muscle strength, the bimanual pole test, and all unilateral movement and dexterity tests—except for the 9-hole peg test, were included in the first factor labelled ‘manual and gross motor skills.’ The second factor consisted solely of the two arm stability measures, which reflects the lack of association between these measures and the remaining upper limb PPA tests. The sensation and fine motor control factor comprised the tests of tactile sensitivity, position sense, 9-hole peg test and finger tapping—the latter a variable also shared with the first factor, while the fourth factor comprised the two tests of tactile discrimination thresholds. This analysis provides insight into the future subgrouping and refinement of tests and assist in the development of a short version with fewer tests.

### Study strengths and limitations

The strengths of this study include the broad selection of sensorimotor tests, the large sample aged across the adult lifespan without diagnosed neurological or musculoskeletal disease and the reliability and validation analyses. We also acknowledge certain limitations. First, given the large number of daily tasks require the coordinated use of both upper extremities concurrently (for review, see [66]), inclusion of additional tests of bimanual function would have provided a more comprehensive model of upper limb function. Second, the inclusion of a test of sensory vibratory sensitivity and discrimination would also have complemented the assessment battery. Third, some data were not collected for the arm stability test, and in particular for men aged 70–79 years. This was due to a synchronization error between the inertial measurement unit worn by the participants and the recording software, and could therefore be considered a non-systematic data loss. For the remaining tests, missing data were few and unlikely to have any major effect on the reported values, especially as the reference scores are reported as medians and percentiles. Fourth, while we recruited participants from a variety of sources, we did not randomly sample from the general population. It is therefore possible that our sample, comprising volunteers, may have been above average with respect to health and fitness. Fifth, we did not assess inter-rater reliability. However, the fact that all tests required only simple instructions and standardised scripts were used is likely to have mitigated against between-examiner test administration variations. Finally, as the sample comprised only neurologically healthy people, further research in clinical groups with neurological impairments is required to determine the thresholds for clinically important differences in the upper limb PPA scores.

## Conclusion

This study provides normative values for upper limb sensorimotor and functional tasks. The tests mostly showed good-to-excellent test-retest reliability, good external validity with respect to age and functional performance, as well as good criterion validity in relation to self-reported upper limb function in those aged 60 years and over. This profiling approach provides a reference range for clinical groups with upper limb sensory and motor impairments and may assist in identifying undiagnosed deficits in the general population.

## Supporting information

S1 Fig Apparatus specifications for two-line discrimination test. (JPG)

S2 Fig Apparatus specifications for bimanual pole test. (JPG)

S1 Table Rationale behind inclusion of selected tests. (DOCX)

S1 File MATLAB code used to calculate total path and example sensor data. (ZIP)

S2 File Data file. (XLSX)
