# Supplementary figures and images for: The upper limb Physiological Profile Assessment: Description, reliability, normative values and criterion validity
Source: PLoS One. 2019 Jun 27;14(6):e0218553. doi: 10.1371/journal.pone.0218553 (PMC6597070; doi:10.1371/journal.pone.0218553)

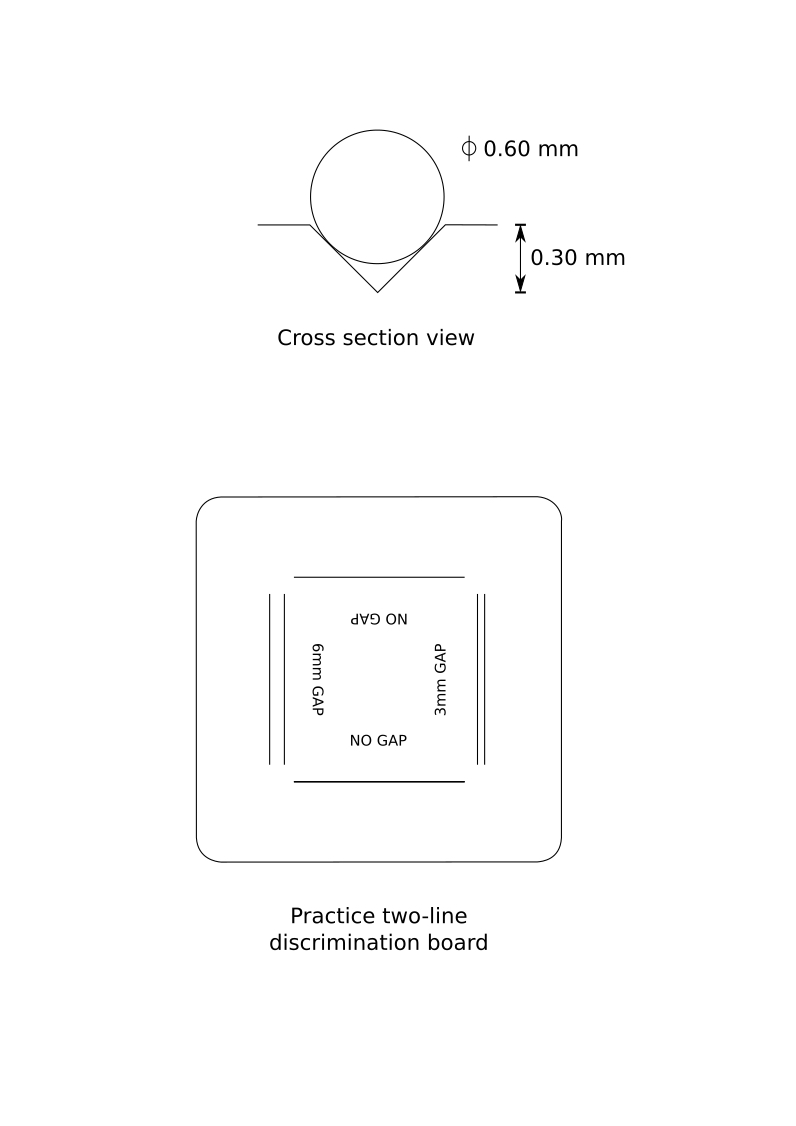

Supplement: S1 Fig — (JPG) [file pone.0218553.s001.jpg]

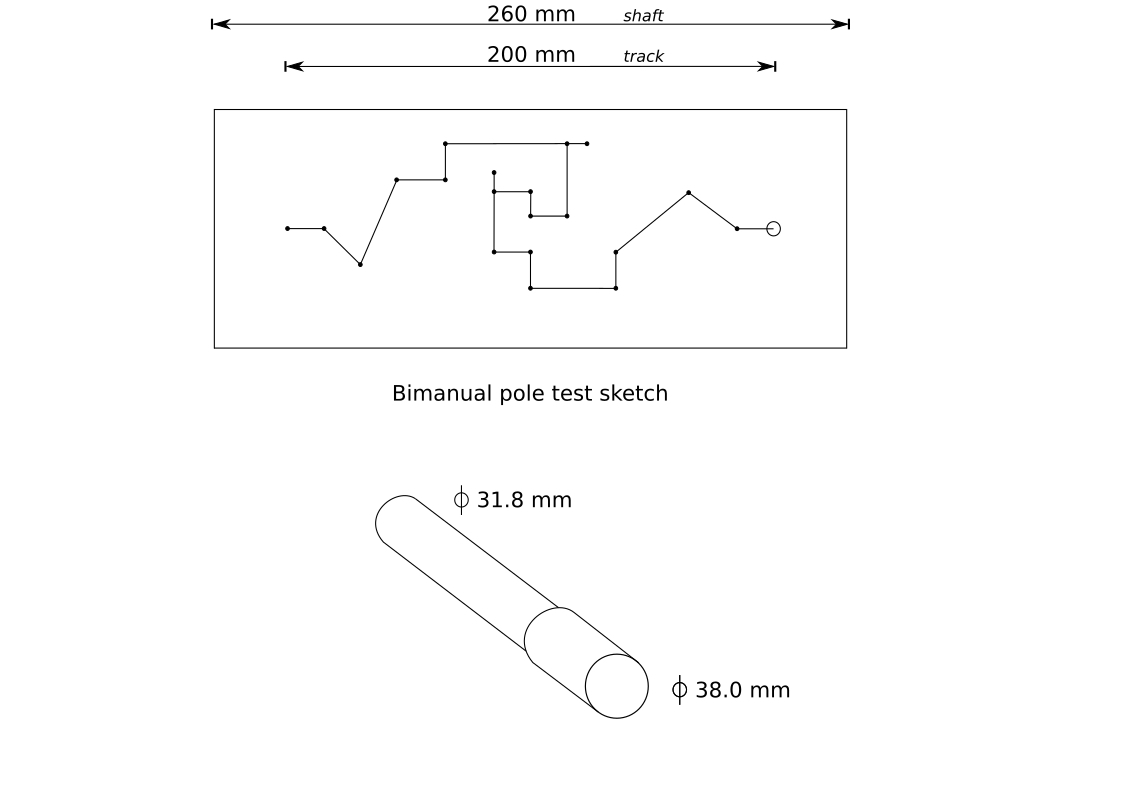

Supplement: S2 Fig — (JPG) [file pone.0218553.s002.jpg]
