# Supplementary material for: The upper limb Physiological Profile Assessment: Description, reliability, normative values and criterion validity
Source: PLoS One. 2019 Jun 27;14(6):e0218553. doi: 10.1371/journal.pone.0218553 (PMC6597070; doi:10.1371/journal.pone.0218553)
Supplement: S1 Table — (DOCX) [file pone.0218553.s003.docx]

| **Physiological system** | **Test** | **Novel** | **Rationale for inclusion** |
| --- | --- | --- | --- |
| Muscle strength | Isometric elbow flexion strength | Adapted [1] | Sufficient upper limb strength necessary to perform ADLs [2,3].  Weakness correlates with functional motor performance in stroke patients [4].  Required for fundamental roles of reach, grasp and manipulation [5].  Need to consider proximal and distal muscle strength, as they are not always proportionally affected in people with neurological pathology [6]. |
|  | Handgrip strength | Validated [7] | See above. |
| Unilateral movement and dexterity | Finger-press reaction time | Validated [8] | Reaction time tests provide a simple means to assess the speed and efficiency of central processing in the brain [9] required for many upper limb motor tasks. An increase in reaction time suggests a compromise in information processing, and is commonly observed in the elderly [10] and in people with neurological conditions such as stroke [11]. |
|  | Finger tapping | Adapted [12] | Provides insight into speeded movements.  Can differentiate patients with mild traumatic brain injury from healthy controls [13].  Used extensively to assess upper limb motor performance in patients with Parkinson’s disease and stroke [14,15]. |
|  | 9-hole peg test | Validated [14] | Considered the gold standard metric for manual dexterity [16].  Manual dexterity is critical to upper limb function, specifically for grasping and manipulating objects. |
|  | Loop & wire test | Custom made | 9-hole peg test does not measure the reaching component of dexterity provided by the proximal segment of the upper limb.  Loop & wire test may provide a more global measure of dexterity.  Offers an alternative assessment in those with severe hand limitations, in which difficulties obtaining the fine motor control required to grasp the individual pegs would otherwise lead to a floor effect in the 9-hole peg test. |
| Position sense | Position sense | Adapted [17] | Proprioceptive acuity is fundamental to the initiation and control of coordinated voluntary movements, therefore plays a critical role in upper limb function. |
| Skin sensation | Tactile sensitivity | Validated [18] | Sensory receptors located within the skin of the hand and fingers are vital in providing immediate feedback through tactile interaction with the environment, a prerequisite for fine motor control [19].  von-Frey filaments are widely recognised as the gold standard for measuring tactile sensitivity [18]. |
|  | Two-point discrimination | Validated [20] | Measures tactile spatial acuity, a vital component of fine motor control [20]. |
|  | Two-line discrimination | Custom made | The reliability and sensitivity of the two-point discrimination test as a measure of tactile spatial acuity has been questioned [21]. |
| Bimanual coordination | Bimanual pole test | Custom made | Many everyday activities require the concurrent use of both upper extremities.  No consensus on a gold standard measure of bimanual coordination that is simple and quick to administer [22,23]. |
| Arm stability | Arm stability test | Custom made | Derived from its lower extremity equivalent in the postural sway test – a strong predictor of falls risk [24].  This test will investigate whether the measurement of arm stability is justified as an essential component of upper limb function. |
| Functional performance | Shirt task | Adapted [25,26] | Provides a potential composite measure of upper limb function encompassing various domains including skin sensation, bimanual coordination and manual dexterity. |

References:

1. Kotte SHP, Viveen J, Koenraadt KLM, The B, Eygendaal D. Normative values of isometric elbow strength in healthy adults: a systematic review. *Shoulder Elbow*. 2018;10:207–215.
2. Harris JE, Eng JJ. Paretic upper limb strength best explains arm activity in people with stroke. *Phys Ther*. 2007:87;88–97.
3. Eng JJ. Strength training in individuals with stroke. *Physiother Can*. 2004:56;189–201.
4. Sommerfeld DK, Gripenstedt U, Welmer AK. Spasticity after stroke: an overview of prevalence, test instruments, and treatments. *Am J Phys Med Rehabil*. 2012:91;814–820.
5. Feys P, Lamers I, Francis G, Benedict R, Phillips G, LaRocca N, Hudson LD, Rudick R. The Nine-Hole Peg Test as a manual dexterity performance measure for multiple sclerosis. *Mult Scler J*. 2017:23;711–720.
6. Lamers I, Kelchtermans S, Baert I, Feys P. Upper limb assessment in multiple sclerosis: a systematic review of outcome measures and their psychometric properties. *Arch Phys Med Rehabil*. 2014:95;1184–1200.
7. [Roberts HC](https://www.ncbi.nlm.nih.gov/pubmed/?term=Roberts%20HC%5BAuthor%5D&cauthor=true&cauthor_uid=21624928), [Denison HJ](https://www.ncbi.nlm.nih.gov/pubmed/?term=Denison%20HJ%5BAuthor%5D&cauthor=true&cauthor_uid=21624928), [Martin HJ](https://www.ncbi.nlm.nih.gov/pubmed/?term=Martin%20HJ%5BAuthor%5D&cauthor=true&cauthor_uid=21624928), [Patel HP](https://www.ncbi.nlm.nih.gov/pubmed/?term=Patel%20HP%5BAuthor%5D&cauthor=true&cauthor_uid=21624928), [Syddall H](https://www.ncbi.nlm.nih.gov/pubmed/?term=Syddall%20H%5BAuthor%5D&cauthor=true&cauthor_uid=21624928), [Cooper C](https://www.ncbi.nlm.nih.gov/pubmed/?term=Cooper%20C%5BAuthor%5D&cauthor=true&cauthor_uid=21624928), [Sayer AA](https://www.ncbi.nlm.nih.gov/pubmed/?term=Sayer%20AA%5BAuthor%5D&cauthor=true&cauthor_uid=21624928). A review of the measurement of grip strength in clinical and epidemiological studies: towards a standardised approach. *Age Ageing*. 2011:40;423–429.
8. Lord Sr, Menz HB, Tiedemann A. A physiological profile approach to falls risk assessment and prevention. *Phys Ther*. 2003:83;237–252.
9. Maes C, Gooijers J, Orban de Xivry JJ, Swinnen SP, Boisgontier MP. Two hands, one brain, and aging. *Neurosci Biobehav Rev*. 2017:75;234–256.
10. Welford AT. Motor performance. In J. E. Birren and K. W. Schaie (Eds.), *Handbook of the Psychology of Aging*. New York: Van Nostrand Reinhold; 1977:450–496.
11. Korner-Bitensky N, Mayo NE, Kaizer F. Change in response time of stroke patients and controls during rehabilitation. *Am J Phys Med Rehabil*. 1990:69;32–38.
12. Hubel KA, Reed B, Yund EW, Herron TJ, Woods DL. Computerized measures of finger tapping: effects of hand dominance, age, and sex. *Percept Mot Skills*. 2013:116;929–952.
13. Prigatano GP, Borgaro SR. Qualitative features of finger movement during the Halstead finger oscillation test following traumatic brain injury. *J Int Neuropsychol Soc*. 2003:9;128–133.
14. Jobbágy A, Harcos P, Karoly R, Fazekas G. Analysis of finger-tapping movement. *J Neurosci Methods*. 2005:141;29–39.
15. Heller A, Wade DT, Wood VA, Sunderland A, Hewer RL, Ward E. Arm function after stroke: measurement and recovery over the first three months. *J Neurol Neurosurg Psychiatry*. 1987:50;714–719.
16. Feys P, Lamers I, Francis G, Benedict R, Phillips G, LaRocca N, Hudson LD, Rudick R. The Nine-Hole Peg Test as a manual dexterity performance measure for multiple sclerosis. *Mult Scler J*. 2017:23;711–720.
17. [De Domenico G](https://www.ncbi.nlm.nih.gov/pubmed/?term=De%20Domenico%20G%5BAuthor%5D&cauthor=true&cauthor_uid=3556473), [McCloskey DI](https://www.ncbi.nlm.nih.gov/pubmed/?term=McCloskey%20DI%5BAuthor%5D&cauthor=true&cauthor_uid=3556473). Accuracy of voluntary movements at the thumb and elbow joints. *Exp Brain Res*. 1987:65;471–478.
18. Dannenbaum RM, Michaelsen SM, Desrosiers J, Levin MF. Development and validation of two new sensory tests of the hand for patients with stroke. *Clin Rehabil*. 2002:16;630–639.
19. McNulty PA, Turker KS, Macefield VG. Evidence for strong synaptic coupling between single tactile afferents and motoneurons supplying the human hand. *J Physiol*. 1999:518;883–893.
20. Dellon AL, Mackinnon SE, McDonald Crosby, P. Reliability of two-point discrimination measures. *J Hand Surg Am*. 1987:12;693–696.
21. Lundborg G, Rosen B. The two-point discrimination test – time for a re-appraisal*? J Hand Surg Eur*. 2004:29;418–422.
22. Maes C, Gooijers J, Orban de Xivry JJ, Swinnen SP, Boisgontier MP. Two hands, one brain, and aging. *Neurosci Biobehav Rev.* 2017:75;234–256.
23. Krehbiel L, Kang N, Cauraugh JH. Age-related differences in bimanual movements: A systematic review and meta-analysis. *Exp Gerontol*. 2017:98;199–206.
24. Lord SR, Clark RD, Webster IW. Postural stability and associated physiological factors in a population of aged persons. *J Gerontol*. 1991:46;M69–76.
25. Chen CL, Yeung KT, Bih LI, Wang CH, Chen MI, Chien JC. The relationship between sitting stability and functional performance in patients with paraplegia. *Arch Phys Med Rehabil*. 2003:84;1276–1281.
26. Boswell-Ruys CL, Sturnieks DL, Harvey LA, Sherrington C, Middleton JW, Lord SR. Validity and reliability of assessment tools for measuring unsupported sitting in people with spinal cord injury. *Arch Phys Med Rehabil*. 2009:90;1571–1577.
